# Genetic inference of on-target and off-target side-effects of antipsychotic medications

**DOI:** 10.1371/journal.pgen.1011793

**Published:** 2025-07-28

**Authors:** Andrew R. Elmore, Aws Sadik, Lavinia Paternoster, Golam M. Khandaker, Tom R. Gaunt, Gibran Hemani

**Affiliations:** 1 NIHR Bristol Biomedical Research Centre, University Hospitals Bristol and Weston NHS Foundation Trust and University of Bristol, Bristol, United Kingdom; 2 MRC Integrative Epidemiology Unit (IEU), Bristol Medical School, University of Bristol, Bristol, United Kingdom; 3 Centre for Academic Mental Health, Population Health Sciences, University of Bristol, Bristol, United Kingdom; University of Michigan, UNITED STATES OF AMERICA

## Abstract

It is often difficult to ascertain whether patient-reported side-effects are caused by a drug, and if so, through which mechanism. Adverse side-effects are the primary cause of antipsychotic drug discontinuation rather than poor efficacy. Using a novel method combining genetic and drug binding affinity data, we investigated evidence of causal mechanisms for 80 reported side-effects of 6 commonly prescribed antipsychotic drugs which together target 68 receptors. We analysed publicly available drug binding affinity data and genetic association data using Mendelian randomization and genetic colocalization to devise a representative ‘score’ for each combination of drug, side-effect, and receptor. We show that 36 side-effects are likely caused by drug action through 30 receptors, which are mainly attributable to off-target effects (26 off-target receptors underlying 39 side-effects). This method allowed us to distinguish which reported side-effects have evidence of causality. Of individual drugs, clozapine has the largest cumulative side-effect profile (Score = 57.5, SE = 11.2), and the largest number of side-effects (n = 36). We show that two well-known side-effects for clozapine, neutropenia and weight change, are underpinned by the action of *GABA* and *CHRM3* receptors respectively. Our novel genetic approach can map side-effects to drugs and elucidate underlying mechanisms, which could potentially inform clinical practice, drug repurposing, and pharmacological development. Further, this method can be generalized to infer the on-target and off-target effects of drugs at any stage of the drug development pipeline.

## Author summary

Many people stop taking antipsychotic medications, not because they don’t work, but because of their side-effects. However, it’s often unclear whether these side-effects are truly caused by the drug. To tackle this, we developed a new method that combines data on how drugs interact with specific receptors in the body and genetic information from large public datasets. We used this approach to investigate 80 reported side-effects of six commonly prescribed antipsychotic drugs. By linking drug targets with genetic signals, we identified likely biological mechanisms behind 36 side-effects. Interestingly, most of these were due to the drug interacting with unintended (off-target) receptors. Clozapine, a widely used antipsychotic, had the most extensive side-effect profile. We found that its known side-effects like weight gain and reduced white blood cell counts could be explained by its action on specific receptors. Our method helps to pinpoint which side-effects are likely caused by which drug actions, offering insights that could improve drug safety and inform clinical decisions. This framework can also be applied to other medications and stages of drug development to better understand both intended and unintended effects. We also discuss where there are gaps in being able to more accurately model drug side effects.

## Introduction

Schizophrenia is one of the leading causes of disability worldwide [1], despite its relatively low estimated international prevalence of 0.33% to 0.75% [2,3]. Antipsychotic drugs exhibit considerable variations in their pharmacological and clinical impacts, including efficacy and tolerability [4]. Differences in adverse effects are understood to be the main drivers of discontinuation, rather than drug efficacy, therefore the adverse effect profile largely determines antipsychotic choice [5]. While many side-effects are reported to be associated with a drug, it is often difficult to ascertain whether patient-reported side-effects are caused by a drug, and if so, through which mechanism. In this study we aim to identify the molecular pathways that are responsible for the side-effect profiles for six major antipsychotic medications.

**Fig 1:**
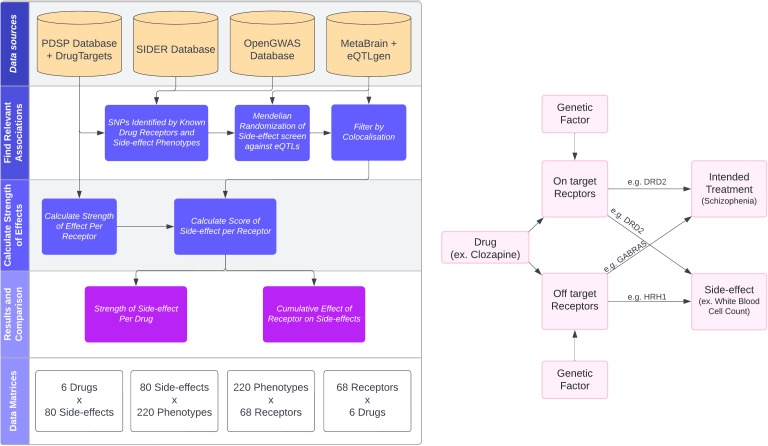
Flowcharts of the methodological processes for predicting drug side-effects. Left: Flowchart of the data sources and methodological steps used to perform the analysis. Right: Directed acyclic graph illustrating how genetic factors are used to predict drug influences on traits via on- and off-target receptors. PSDP: Psychoactive Drug Screening Program; SIDER: Side-effect Resource; GWAS: Genome Wide Association Study; SNP: Single Nucleotide Polymorphism; eQTL: Expression Quantitative Trait Loci.

Both beneficial and adverse effects can be attributed to differences in target receptors and binding affinities among antipsychotics [4,6]. For example, clozapine is efficacious for those with treatment-resistant schizophrenia, is the only FDA-approved treatment for treatment-resistant schizophrenia [7] and has a unique side-effect profile, reported to cause neutropenia and more commonly cause weight gain [8]. This unique side-effect profile compared to other antipsychotics is likely due to differences in the receptors it binds, and differences in the affinity with which it binds them (Fig 1). The most common receptor targets for antipsychotics are in the dopamine and serotonin families [9] and there are many off-target receptors for these drugs which vary by drug [10].

Mendelian randomization (MR) is an established statistical method that can use genetic variants to estimate causal effects [11] of genetically-proxied protein abundance on the outcome of interest. The main advantage MR has over traditional observational epidemiological methods is that MR can imply causality between an exposure and an outcome because it is less liable to common epidemiological biases, such as confounding and reverse causality. Genetic colocalization provides additional supportive genetic evidence by assessing whether two association signals are consistent with a shared causal variant [12].

**Fig 2:**
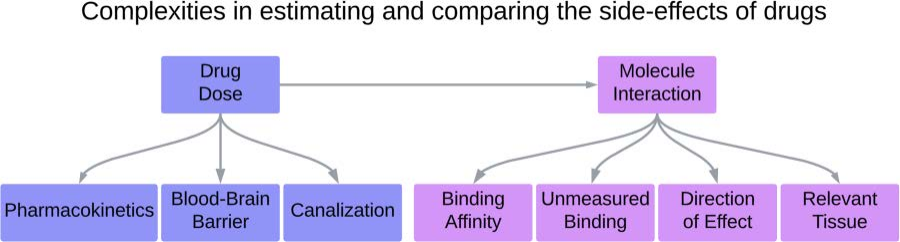
Complexities in predicting the side-effects of drugs. Each block in this figure has specific problems, with our proposed resolutions. Not included in this figure is an additional challenge which is that comparison of predicted to side effects and real world side effects is hindered by lack of high coverage quantitative data on observed side effects or data from trials. **Pharmacokinetics:** Problem: Differential pharmacokinetics by drug can modify the concentration in the relevant tissue. Resolution: Use estimates of DDD to scale effects on the assumption that they capture differential pharmacokinetics. **Blood-Brain Barrier**: Problem: Some drugs have different rates in which they pass through the blood brain barrier. Resolution: An aspect of drug dose requires an adjustment for pKi and passing the blood-brain barrier, so this is also captured in the drug dose scaling. **Canalization**:Problem: Drug effects may be dampened or buffered by compensatory developmental processes. Resolution: It is not directly possible to detect or determine if canalization has occurred, but important to keep in consideration when evaluating results. **Binding Affinity**: Problem: There are different measures of binding affinity, some of which have significantly disparate measurement results. Resolution: Take a trimmed mean of the binding affinity measurements. **Unmeasured Binding**: Problem: Not all receptors are measured for binding affinity, and some receptors have suspected effects. Incorporating these receptors can be important to paint a full picture of drug effects. Resolution: We have decided to provide a default conservative binding affinity value based on other receptor binding affinities. Another solution could involve attempting to estimate based on protein-protein or molecule-protein interaction estimators. **Direction of Effect**: Problem: eQTL direction has been shown differ to protein expression, and some binding effects (like partial agonist) have non-linear binding relationships. Resolution: Report cumulative results without direction of effect, additional corroborating research must be considered before reporting a specific direction. **Relevant Tissue**: Problem: Drugs do not only interact with one tissue in the body, they can affect different systems, of which a single QTL measurement may not capture, especially when comparing using genetic colocalization. Resolution: Use different eQTL datasets to capture the different. We used both MetaBrain and eQTLGen. Rely on pleiotropic eQTL effects which correlate across different tissues.

We have developed a novel method to gain insights into drug side-effects and which receptors contribute to each side-effect, and at what strength (Fig 1). We have done this by integrating gene expression quantitative trait loci (eQTL) data and data on drug-protein binding affinities within a Mendelian randomization framework. There are a number of on-going challenges in this endeavour (Fig 2), but the results serve as a proof of principle for genetically predicting on-target and off-target drug side-effects. This method is not limited to schizophrenia medications and can be applied to any drug that has a range of target receptors and side-effects. Using genetic data to estimate the causal effects of targets on reported side-effects could help inform drug development to reduce side-effects, predict drug side-effect profiles before clinical trials take place or inform prescribing practice for licensed drugs.

While our study leverages tissue-specific eQTL data to explore potential mechanisms underlying antipsychotic side-effects, it is important to note that colocalization signals should not be interpreted as definitive evidence of tissue-specific mechanisms. Many eQTL signals are shared across tissues, and observed associations may reflect pleiotropic effects rather than tissue-specific pathways. We relied on the documented phenomena that cis eQTLs tend to be correlated and colocalizing across different tissues [13].

## Results

Of the 68 receptors that are targeted by the 6 antipsychotic medications, 30 receptors show evidence consistent with a potential causal effect on a reported side-effect, while 38 do not show evidence of a causal effect. Of the 30 receptors, 26 of those were due to off-target effects of the drugs. Three out of the 5 known on-target receptors showed evidence of causing a reported side-effect (*DRD2*, *DRD4*, and *HTR2A*), as well as 1 out of the 3 suspected-target receptors (*HRH3*).

Of the 80 side-effects that were proxied by GWAS traits, we found 44 that appear to be causally influenced by at least one receptor that binds an antipsychotic drug. Of these 44 side-effects, 36 are predominantly influenced by off-target receptors (> 50% of cumulative score), while 8 are predominantly caused by on-target receptors.

**Fig 3:**
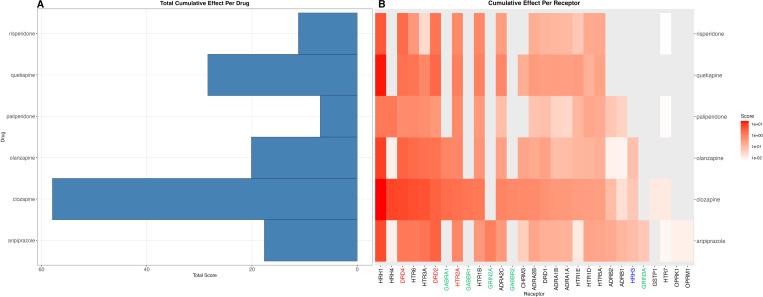
Cumulative Effect Score per Drug. A: Total Cumulative Effect Per Drug. Each side-effect score per receptor is summed per drug, showing the cumulative side-effect profile per drug, and the receptor that causes it. Red receptor name signifies on-target receptors, blue receptor name signifies suspected-target receptors, green signifies unknown ki values of the receptor. B: Breakdown of drug scores by receptors. Each side-effect score is summed per receptor, showing the cumulative side-effect profile per receptor across all side-effects. Red names signify on-target receptors, blue receptor name signifies suspected-target receptors, green signifies unknown ki values of the receptor. Receptors in red signify on-target receptors, receptors in green signify suspected-target receptors, while side-effects in black signify off-target receptors.

Clozapine has the largest cumulative side-effect profile (Score = 27.4, SE = 5.2), as well as the largest number of side-effects (n = 46), showing it has the strongest and most varied side-effect profile of the 6 drugs (Fig 3).

### Breakdown of side-effects for on-target and off-target receptors

**Fig 4:**
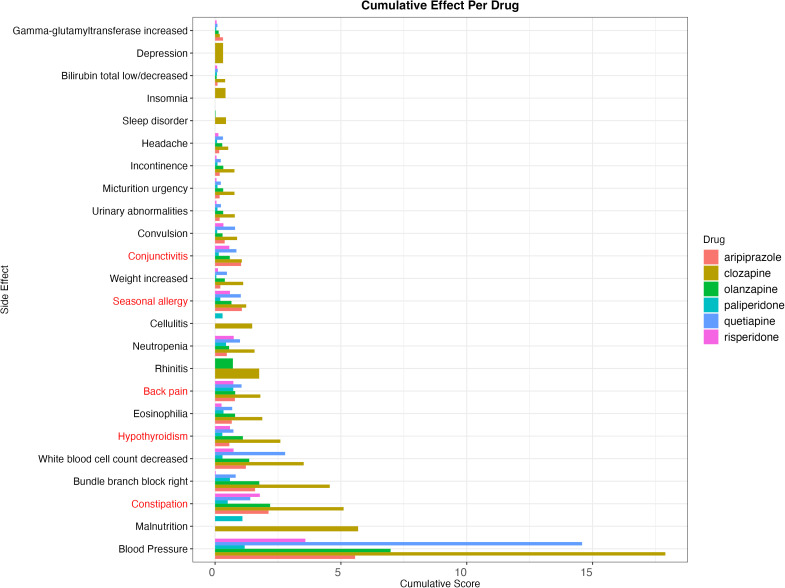
Breakdown of drug scores by side-effect. Each receptor score is summed per side-effect, showing the cumulative side-effect score across all receptors. Side-effects in red signify evidence of bring primarily caused by on-target or suspected-target receptors (> 50% of cumulative score), while side-effects in black signify evidence of bring primarily caused by off-target receptors.

The off-target receptors with causal evidence on side-effects are the histamine receptors (*HRH1*, *HRH4*), serotonin receptors (*HTR6*, *HTR3A*, *HTR1B*, *HTR5A*, *HTR1E*), adrenergic receptors (*ARDA2C*, *ADRA2B*, *ADRB1*, *ADRB2*, *ADRA1A*, *ADRA1B*), and *GABA* receptors (*GABRA1*, *GABBR2*, *GABBR1*). GABA receptors are only bound by the drugs clozapine and olanzapine (Fig 4).

The receptors for which we found causal evidence of 4 or more side-effect traits are *DRD2* [11], *GABBR1* [6], *HTR6* [8], *GABRA1* [4], *HTR1D* [4], and *HTR1E* [4] (Fig C in S1 Text).

There are 28 side-effects that show evidence of being predominantly caused by off-target receptors (> 50% of the total score), which include blood pressure and neutrophil count, and 8 side-effects that show evidence of bring primarily caused by on-target or suspected-target receptors, which include constipation and hypothyroidism (Fig 4).

Clozapine and olanzapine are distinct from the other drugs in binding to members of the *GABA* receptor family, which has been speculated to influence schizophrenia via the *GABRA5* receptor through upstream regulation of *DRD2* [9]. *GABA* receptors do not have binding affinity data in PDSP, so the default value was used for calculation, therefore the magnitude of results may be different than reported. Many side-effects linked to *GABRA1* are immune related: upper respiratory tract infection and neutrophil count, white blood cell count, and rhinitis.

### Mechanisms for specific side-effects

**Fig 5:**
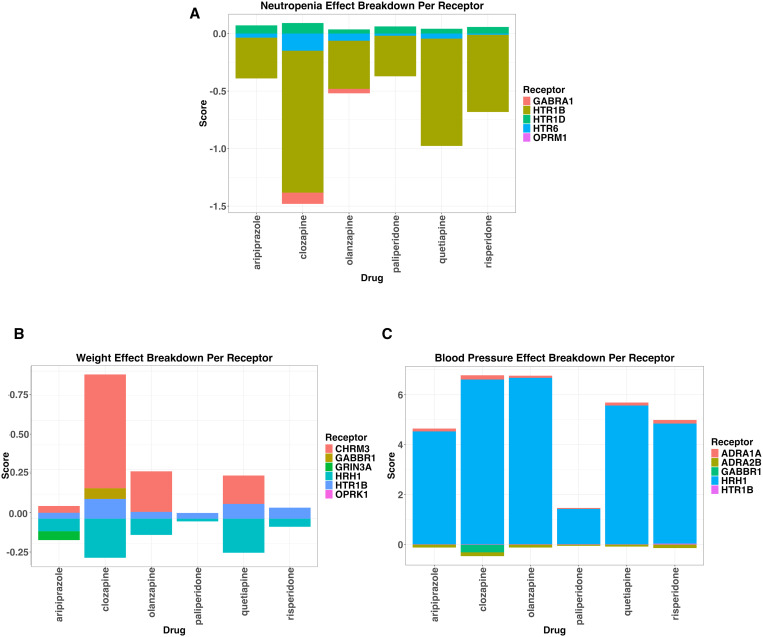
Breakdown of Specific Side-effects of Interest per Drug. Each side-effect score is broken down per receptor, with direction indicating the relationship of the MR result of eQTL gene expression compared to phenotype, corrected by binding affinity direction. eQTL: Expression Quantitative Trait Loci. A: Scores of ‘Neutropenia’ per drug, broken down by receptor and direction of effect. B: Scores of ‘Weight increase’ per drug, broken down by receptor and direction of effect. C: Scores of ‘Blood Pressure’ per drug, broken down by receptor and direction of effect.

We have focussed on 3 different side-effects due to their severity, their effect and what our results can highlight about the side-effect (Fig 5). We have also compared our results to existing literature with regards to severity and prevalence (Table 1).

**Table 1:** Comparison of Side-effect of side-effect frequency vs. study results.

	Side-effect	ARI	CLO	OLA	PAL	QUE	RIS
Existing Literature	Weight Gain	+	++++	+++	+++	+++	+++
Results (Score)	Weight Gain	0.21	1.12	0.38	0.05	0.47	0.11
Existing Literature	Neutropenia	+	++++	+	+	+	+
Results (Score)	Neutropenia	0.47	1.59	0.55	0.43	1.00	0.74
Existing Literature	Blood Pressure	++	+++	++	+	+++	++
Results (Score)	Blood Pressure	5.55	17.80	6.93	1.19	14.50	3.57

“Existing Literature” is based on Table 1 from Stroup et. al [31], where categorisations are an amalgamation of severity and prevalence. “Results” are the absolute value of scores, aggregated by side-effect and drug.

+: minimal/rare, ++: mild/sometimes occurs, +++: moderate/occurs frequently, ++++: severe/occurs very often.

ARI: aripiprazole, CLO: clozapine, OLA: olanzapine, PAL: paliperidone, QUE: quetiapine, RIS: risperidone.

### Neutropenia

Neutropenia (low white blood cell count) is a known rare adverse side-effect of clozapine [14] and olanzapine [15], which is of higher concern for clozapine. Our findings show the more severe neutropenia effects may come from the targeting of GABRA1 by clozapine (*GABRA1* score = 0.09, SE = 0.01) and olanzapine (*GABRA1* score = 0.03, SE = 0.01) which is not targeted by other drugs, with an additional larger effect of *HTR1B* for clozapine (score = 1.24, SE = 0.27). Due to a lack of binding affinity data for GABA receptors, it is difficult to predict exactly how much of an effect those targets may have, or to compare cumulative effects across drugs.

### Weight gain

Antipsychotic-induced weight gain is a key concern for patients, and common cause of discontinuation [16]. Clozapine and olanzapine have previously been reported as having a higher risk of weight gain [16], which is corroborated by our results (Fig 5). The higher binding affinity of clozapine to *CHRM3* (Score = 0.69, SE = 0.09), *HRH1* (Score = 0.24, SE = 0.03), *HTR1B* (Score = 0.12, SE = 0.02), and *GABBR1* (Score = 0.06, SE = 0.01, specifically in clozapine) appear to cause the difference in weight gain between those two drugs and others, and clozapine has the largest score against weight gain.

### Blood pressure

The effects of antipsychotics on blood pressure remain unclear: some studies suggest there are links to hypertension across atypical antipsychotics [17], and others suggest a link to hypotension across antipsychotics, with some reports of hypertension [18]. Our results show that antipsychotics affect blood pressure in potential differing directions of effects, given the direction of the QTL associations based on the receptor. The large scores for blood pressure are driven by HRH1, which has both high binding affinities and Wald ratios for systolic blood pressure.

Additional bar charts of pertinent results broken down by side-effect (Fig A in S1 Text), and by receptor (Fig B in S1 Text). All score results are provided (Table A in S1 Tables).

## Discussion

Our novel method allows us to use genetic data to unpick the mechanism of action that a drug has on a specific side-effect, and further allows us to compare the size of those effects between drugs. Our results closely corroborate many known side-effect profiles and explains their occurrence due to specific targets and differential target-binding between drugs. This approach could also be used to help inform drug choice in patients, as many patients try different antipsychotic medications before finding an appropriate balance between efficacy and tolerability [19]. As an example, our evidence shows that the side-effect urinary incontinence is strongest in clozapine, and if the patient already suffers from it, another drug with evidence of a weaker effect may be preferred, which would improve tolerability and adherence.

The results also inform which drugs have genetic evidence supporting them and are less likely to be caused by other means, for this reason the results can additionally inform which side-effects have should be monitored in future trails.

Understanding which receptors cause side-effects provides an opportunity to counteract the more clinically pertinent side-effects of drugs with multiple targets. Using our results, we have compiled a table of results that highlights the receptors that are likely leading causes of side-effects, which is corroborated by existing literature, paired with existing medications which are prescribed for other treatments that target the highlighted receptor (s) in question (Table 2). These results highlight both which receptors may cause side-effects (*CHRM3* for weight, GABA for neutropenia, histamine for blood pressure, weight, and white blood cell count), as well as which existing drugs may work to combat these effects. Additional consideration to drug interactions should be applied before prescribing. We cross-referenced the suggested drugs in Table 2 with the British National Formulary (BNF) interactions list (https://bnf.nice.org.uk/interactions/). Of which only Brimonidine was noted as potentially increasing hypotension along with the 6 antipsychotics. However, as discussed, the antipsychotics may cause hypertension or hypotension and would be prescribed for those who see an increase of blood pressure.

**Table 2:** Table of Highlighted Side-effects, and Potential Drugs That Could Counter Specific Effects.

Receptor	Found Side-effect	Existing Literature	Drugs that act on receptor in other direction
CHRM3 Antagonist	Weight	CHRM3 and Weight [32]	**Cevimeline**: acts on CHRM3 as an agonist. Showed to partially reverse effects of olanzapine in rats, including weight gain.
HRH1, HRH2, HRH3, HRH4 Antagonists	Blood pressure, weight, white-blood cell, gamma-glutamyltransferase levels	HRH1 and Weight [33] HRH1 and White-blood cell count decrease [34] HRH1 and Hypertension [35,36]	**Histamine**: acts on HRH1–4 as an agonist.
GABBR1 Positive modulator	Weight, neutropenia, blood pressure	GABBR1 and Blood stem cells [37] GABA receptors and Weight [38] GABBR1 and blood pressure [39]	**Acamprosate:** inhibitor for GABBR1, and acts on GRIN receptors.
ADRA2A, ADRA2B, ADRA2C Antagonists	Hypertension	ADRA2B: Blood pressure, cardiovascular function, lipid metabolism [40]	**Brimonidine:** acts on ADRA2A-C as an agonist.

Further, this approach could be used to investigate any drug that targets a wide range of receptors and has a wide range of reported side-effects, leading to novel insights across a range of diseases.

There are examples of results that are inconsistent with existing literature. For example, risperidone has a higher overall score for neutrophil count, due to high affinity to *HTR1B* and a lack of binding affinity data for the *GABA* receptors, for which the default Ki values were used, and may not be accurate. Further, constipation results do not reflect clinical data, namely clozapine is known to cause fatal gastric hypomotility [20]. This may be due to binding to opioid receptors that are also not captured in Ki databases. Further explanation on the restrictions to this analysis is discussed in limitations.

## Limitations

The limitations of this analysis fall into two primary categories: first, the complexities involved in estimating and comparing the side-effects of drugs; and second, the constraints related to the availability of data.

Regarding complexities involved in estimating and comparing side-effects of drugs, we have outlined a series of challenges and resolutions to each potential limitation (Fig 2).

Regarding constraints arising in the availability of data, we encountered the following difficulties. We were limited first by incomplete binding affinity data for each drug. Some binding affinity data is not available for known or suspected-target receptors, and there may be more receptors that have not been measured which could further contribute to side-effects (Table C in S1 Tables). Reported binding affinity data comes from a variety of labs, potentially using different protocols, and with differing potential measurement errors. Reported binding affinity data per receptor and drug found in PDSP and DrugBank occasionally have a large variance; using a trimmed mean to choose the binding affinity value is helpful to remove the most egregious outliers, and provides a standard error, which could be used for parametric bootstrapping when calculating the score.

Second, we were limited by the availability of GWAS data. Some side-effects might not have a corresponding phenotype measured in a population study, and some side-effects might not have a genetically-caused equivalent. The list of side-effects that can be investigated using genetic data is dependent on a GWAS of that phenotypic trait being available.

Third, the directionality of effect is liable to be incorrect. Using MR via the Wald ratio and taking the drug target action into account could provide for an understanding of whether each association was protective or deleterious. However, the MR results show a direction of effect against the MetaBrain eQTL, estimates of eQTL and pQTL effect direction have been shown to disagree for 30% of genes [21]. Although pQTL measurements would likely be a better proxy for receptor binding of a gene, data are currently lacking. Only 2 out of the 68 genes of interest exist in the UK Biobank Pharma Proteomics Project (UKB-PPP), therefore eQTLs were preferred. Additional care would also need to be made with the function of partial agonists, which are present in clozapine and aripiprazole. Further, the relationship between receptor abundance and agonism is not straightforward and depends on the availability of the ligand or whether other processes are blocking the receptor. Additionally, MR effect estimates represent a lifetime exposure to a low ‘dose’ of perturbed receptor levels, which does not necessarily predict the intervention on those receptors due to acute pharmacological treatment in adulthood.

Fourth, there are limitations in filtering by genetic colocalization depending on the eQTL dataset. Gene expression can vary widely based on the tissue that they are expressed in. An eQTL dataset based on assays from brain tissue may not capture the effect of the gene expression in other tissues or cell types relevant to the disease. Both MetaBrain and eQTLgen were used to capture some of the variety of gene expression, but it will not capture all variety.

Finally, the calculated score, which combines MR results and binding affinity data per receptor, may not translate directly to the clinical significance of strength of effect of the side-effect in question. This is especially true when any score calculation has included default binding affinity data, or data with a wide standard error. Further, it is not clear how the score reflects the severity of the side-effect. There is a relevant correlation between scores and side-effects when comparing side-effect scores between drugs, however, comparing scores between side-effects does not yield any discernable pattern. Therefore the scores seem to be most useful when comparing drugs.

The analysis is also limited by ancestry. MetaBrain is a European only dataset, and eQTLGen is a majority European dataset. Although some of the GWAS summary statistics that were used from OpenGWAS were multi-ancestry, all had the majority of the samples used from European individuals and therefore the results of the GWAS were dominated by European individuals. Consequently, the analysis is internally consistent to provide relevant results, but we cannot generalize our findings to individuals from other ancestries.

Whilst the proposed approach appears to provide novel insight into mechanisms of drugs and potential side effects beyond what is typically available at the point of target selection in drug discovery pipelines, the results are insufficient to provide concrete clinical translation guidance. However, adopted into drug discovery pipelines, we believe this approach can serve as a useful early indicator of the potential side effect profile of drugs in early development.

## Methods

### Identifying drug targets and side-effect phenotypes

We focussed on six commonly prescribed antipsychotic drugs, including clozapine, which has the highest overall efficacy [4]: aripiprazole, clozapine, olanzapine, paliperidone, quetiapine, and risperidone. These drugs bind to a total of 68 target receptors based on information from two sources: The Psychoactive Drug Screening Program (PDSP) Ki database [10], and the DrugBank database [22]. The six drugs have 165 different side-effects associated with them, based on a reported lower bound frequency of 1% in the Side-effect Resource (SIDER) database [23]. Of the 165 side-effects, we were able to genetically proxy 80 with genome-wide association study (GWAS) data for a corresponding phenotype in the OpenGWAS repository [24] and standardise the data by creating unique identifiers across data sources and converging differing effect score calculations [25]. There is some redundancy in our mapping of side-effects to GWAS phenotypes, resulting in the 80 side-effects being mapped to a total of 220 related phenotypes with GWAS results (Table B in S1 Tables).

The *on-target receptors* which underly antipsychotic treatment responses belong to the dopamine (*DRD2*, *DRD3*, and *DRD4*) and serotonin families (*HTR2A* and *HTR2C*) [9]. Other receptors have some evidence of influencing schizophrenia symptoms, but they are not intentionally targeted, and their clinical evidence is not as strong. These receptors, which will be referred to as *suspected-target receptors*, include the *GABA* family (specifically *GABRA5*), which shows upstream regulation of *DRD2*, and the histamine family (specifically *HRH2* and *HRH3*) [9].

### Genetic instrument identification for side-effect phenotypes

In order to evaluate the potential causality between a drug and reported side-effects, we estimated the influence of perturbing each protein that the drug binds with. Our approach of using Mendelian randomization (MR) to test potential causal effects of proteins on a range of phenotypes requires each protein to have a genetic instrument – a variant, usually a single nucleotide polymorphism (SNP), that strongly associates with the protein level in a relevant tissue.

To identify genetic instruments, we first selected genes from existing literature on the receptors that the drugs bind to (using both PDSP and Drugbank databases). Instrumental variables were then chosen based off of the top hits as found in the eQTL datasets for each corresponding gene. We used the MetaBrain [26] and eQTLgen [27] eQTL datasets. This resulted in 1 SNP for eQTLGen, and between 1 and 4 SNPs from MetaBrain, depending on if each brain region had a different top hit. We used 5 brain regions (basal ganglia, cerebellum, cortex, hippocampus, and spinal cord). The list of SNPs corresponding to each brain region is supplied in the supplementary material (Table D in S1 Tables).

Only *cis*-acting genetic variants were used, which is classified as those located near (within ±1Mb) the gene’s regulatory region, and as *trans*-acting when located outside this window. We did not include *trans****-***acting variants because MetaBrain only reports the statistically significant *trans*-eQTLs without information about their surrounding regions [26], therefore performing genetic colocalization analyses for the *trans*-acting variants is not possible.

### Side-effect screening analysis

To obtain a profile of downstream consequences of perturbing each of the drug binding proteins, we ran a “side-effect screening” against each instrument using the OpenGWAS platform. This involved extracting genetic associations from published GWAS summary statistics for each genetic instrument (for each protein). We then filtered the results against the 220 phenotypes selected. To account for multiple testing, p-values were filtered using a false discovery rate (FDR) threshold < 0.05, and results were then filtered to retain only one result for each side-effect/receptor pair.

### MR and genetic colocalization analyses of drug target proteins and side-effect phenotypes

For each drug target and side-effect phenotype pair (i.e., protein-trait pair) identified in the previous step, we performed MR to obtain an estimated causal effect of the tissue-specific expression level of the protein on the side-effect phenotype.

MR relies on three assumptions for identifying a putative causal effect [28], the genetic instrument should: 1) associate with the exposure (relevance), 2) have no shared causal factors with the outcome (independence), and 3) solely influence the outcome through the impact of the risk factor of primary concern (exclusion restriction).

The relevance assumption was enforced by selecting QTLs that were robustly associated with the molecular trait, this was tested by generating the F-statistic for each instrument, where an F-statistic > 22 is evidence against weak instrument bias [29]. MR estimates were obtained using the Wald ratio method and results were then filtered based on FDR < 0.05.

As our MR analyses are based on a single SNP for each protein, additional steps were needed to strengthen the argument of causal inference for both the independence and exclusion restriction assumptions. Genetic colocalization analysis was used to assess whether two traits (i.e., drug target protein and side-effect phenotype) shared a causal variant [12]. Depending on the phenotype, genetic colocalization was performed against either the MetaBrain eQTL dataset if the side-effect response was likely to be relevant to brain function, or the eQTLgen eQTL dataset [27] otherwise.

Genetic colocalization refers to the sharing of a causal variant between two or more traits. The package *coloc* was used to assess whether two association signals (representing drug target and side-effect phenotype) are consistent with a shared causal variant [12]. We assessed whether the posterior probabilities (PP) of the analysed SNPs represented a shared causal variant (known as “hypothesis 4”, H4), and were not associated with different causal variants (known as H3). We observed that occasionally the colocalization method concluded no shared signal at the standardised threshold of PP > 0.8 due to lack of evidence of the outcome trait having a strong enough association. Hence, we filtered colocalization results requiring evidence of a shared effect to be H4 > 0.6, whilst also requiring evidence for distinct causal effects H3 < 0.1. This is to allow differentiation between colocalization results that have the same causal effects but are less powered (H4), and colocalization results that have some evidence of different causal effects (H3).

Additional explanation on the justification for setting our results to H4 >= 0.6 and H3 < 0.1 can be found in supplemental materials (Text A in S1 Text).

GWAS and QTL datasets are either solely from European ancestry, or in the case of some GWASes are meta-analyses, of which 90% or more of the participants in the GWAS are from European ancestry. Binding affinity data is a cumulation of studies, however most binding affinity cell lines are derived from European populations. Although the analysis does not account for ancestral differences, the data that is used is ancestrally consistent.

### Calculating effect score of side-effect per receptor

Having obtained effect estimates $\beta_{IV,e}$ for each of E binding proteins genetically colocalizing with a side-effect proxying trait, we build a score $S_{d,t}$ to characterise the impact of a drug d on that particular side-effect t,


$$S_{d,t}=\sum_{e}^{E}\left(\beta_{IV,e}\times (pK_{i,e}+a_{d}))\right)$$

where the contribution of each protein to a drug’s profile is a product of its binding


$$pK_{i}=\;-\log_{10}(\overset{―}{K_{i}M})-4$$

$$\beta_{IV}=\;\frac{\beta_{ZY}}{\beta_{ZX}}$$

$$a_{d}=\;{pK_{i,e}\times \log}_{10}\left(d_{e}\right)$$

Where:

- E is a list of MetaBrain genes per drug that pass colocalization with the trait t.
- pKi is a scaled version of the mean binding affinity data, $\overset{―}{K_{i}M}$, such that values are between 0 and 4.5 on the logarithmic scale, and $K_{i}M$ is the inhibition constant.
- $a_{d}$ is the dose equivalent per drug, adjusted to a logarithmic scale by taking the log of the dose equivalence (centered around 1), and adding a scaled version of the pKi to itself. $d_{e}$ is the drug dose equivalent value.
- $\overset{―}{K_{i}M}$ is calculated by converting $M=nM*1e^{-8}\;$ using a trimmed mean threshold = 0.1 of all values in the PSDP Database, in order to ignore extreme outliers.
- $\beta_{IV}$ is defined as the Wald Ratio estimate where $\beta_{ZY}$ is the phenotypic result of the SNP, and $\beta_{ZX}$ is the MetaBrain QTL SNP.

Finally, $S_{d}=\sum_{t}^{T}S_{d,t}$ is the total side-effect score for a drug based on the sum of all per-side-effect scores. Both $\overset{―}{K_{i}M}$ and $\beta_{IV}$ have standard errors, and to obtain the standard error for $S_{d,t}$ we used parametric bootstrap with 1000 iterations. The dose equivalents between antipsychotic medications were taken from existing literature [30]. An example of the algorithm used to create these scores and a walkthrough of how it can be used in a R markdown file can be found at https://github.com/andrew-e/side-effect-score.

Performing this analysis comes with complexities in both estimating and comparing side-effects (Fig 2). If there were no known binding affinity values for a specific ligand, a default value of the mean value of all known binding affinity measurements across the 6 drugs was calculated, which is $pK_{i}$= 0.824 (SE = 0.15). There is no known binding affinity in PDSP for the *GABA* or glutamate families of receptors, among others (Table C in S1 Tables). All binding affinity values ${\geq 10,000\;K}_{i}(nM)$ were discarded as having no substantial effect on the receptor.

Direction of effect was not relied upon due to data limitations. As eQTL data been shown to disagree with corresponding protein QTL effect direction up to 30% of the time [21], the direction of the MR results may not indicate true direction of effect on the drug. Therefore, when investigating the direction of the results, additional corroborating research must be considered. We report results for effects per drug and receptor with direction of effect, but graphs summarising effect per drug or receptor are reported cumulatively. Specific side-effect and receptor combinations were investigated more deeply. The mechanism of action (antagonist vs. agonist) and a phenotype specific adjustment of the GWAS have also been considered when incorporating direction of effect in the reported results.

The results shown and subsequent discussion regarding the possibility of drug repurposing are not intended to be taken as clinical guidance for antipsychotic use. Rather this work is intended to illustrate potential value of our approach to understand mechanisms of drug side effect. We are providing a novel method which provides insights into the mechanisms of drugs which may lead to future pharmacogenomic investigations. The results provided are not immediately translatable for informing personalised drug choices for patients.

## Conclusion

Through our novel method, we have been able to provide a fuller understanding of the known side-effects of commonly prescribed antipsychotics. We have been able to look at which receptors are likely to cause side-effects, and gained the ability to differentiate which side-effects show evidence of being caused by on-target receptors compared to off-target receptors. Through these insights, we are able to gain novel insights in 3 ways.

First, this approach could be used to help inform drug choice for patients and clinicians, by giving clinicians a clearer idea of both drug-specific side-effects, and of which alternative drug (s) may have the same side-effects. This could enable clinicians to avoid switching patients that have suffered a particular side-effect to an alternative that likely has the same side-effect.

Second, predicting which receptors cause which side-effects could help inform novel drug creation to reduce side-effects (by minimising binding to these receptors), as well as predict drug side-effect profiles before clinical trials take place. If binding affinity data were to be collected before clinical trials, the side-effect profiling of drugs could occur early in the process, ensuring adequate data are collected during the trial, thereby improving the efficiency and safety of drug creation.

Third, understanding which receptor is causing an adverse side-effect opens new drug repurposing possibilities for drugs, as shown in Table 2.

## Supporting information

S1 Text Supplemental Text and Figures. **Fig A.** Cumulative side-effects broken down by receptor. Series of bar charts of cumulative side-effect scores reported per drug. The different colours represent a different receptor that the score is attributed to. **Fig B**. Cumulative receptor side-effects per drug. Series of bar charts of cumulative receptor scores reported per drug. The different colours represent a different receptor that the score is attributed to. **Fig C**. Number of side-effects per receptor. Number of effects caused by a specific receptor. Receptor names coloured red are on-target receptors, blue are for suspected on-target receptors, and green are suspected receptors with no binding affinity. **Text A**. Explanation of Colocalization Thresholds. Explanation and additional evidence for the justification of using the suggested colocalization thresholds.
(DOCX)

S1 Tables Supplemental Tables. **Table A**. Scores. Table that combines all relevant information to the GWAS studies used, the side-effect it is associated with, the SNP, wald ratio, colocalization score, source of eQTL and drug. These are the values used to calculate overall scores. **Table B**. Side-effect map. Map used to draw associations between a side-effect (column A) and the corresponding PheWAS trait (column B). **Table C**. Receptor + Ki values. Matrix of receptors to drug binding affinity targets, binding affinity values, and action. Binding affinity information was obtained using the Psychoactive Drug Screening Program (PDSP) Ki database, and the DrugBank database. **Table D**. Exposure SNP Map. Matrix of receptors to SNPs that are associated with that receptor in different regions of the brain, according to MetaBrain.
(XLSX)

S1 STROBE MR Checklist The STROBE-MR checklist contains 20 items recommended to address in reports of Mendelian randomization studies. (DOCX)
