# Supplementary material for: Genetic inference of on-target and off-target side-effects of antipsychotic medications
Source: PLoS Genet. 2025 Jul 28;21(7):e1011793. doi: 10.1371/journal.pgen.1011793 (PMC12321077; doi:10.1371/journal.pgen.1011793)
Supplement: S1 Text — Fig A. Cumulative side-effects broken down by receptor. Series of bar charts of cumulative side-effect scores reported per drug. The different colours represent a different receptor that the score is attributed to. Fig B. Cumulative receptor side-effects per drug. Series of bar charts of cumulative receptor scores reported per drug. The different colours represent a different receptor that the score is attributed to. Fig C. Number of side-effects per receptor. Number of effects caused by a specific receptor. Receptor names coloured red are on-target receptors, blue are for suspected on-target receptors, and green are suspected receptors with no binding affinity. Text A. Explanation of Colocalization Thresholds. Explanation and additional evidence for the justification of using the suggested colocalization thresholds. (DOCX) [file pgen.1011793.s001.docx]

S1 Text: Supplementary Online Content

**Contents:**

**Figure 1**: Cumulative side-effects broken down by receptor

**Figure 2**: Cumulative side-effects per drug across receptors

**Figure 3:** Number of side-effects per receptor

**Text 1:** Explanation of Colocalization Thresholds

## Figure 1: Cumulative side-effects broken down by receptor

| 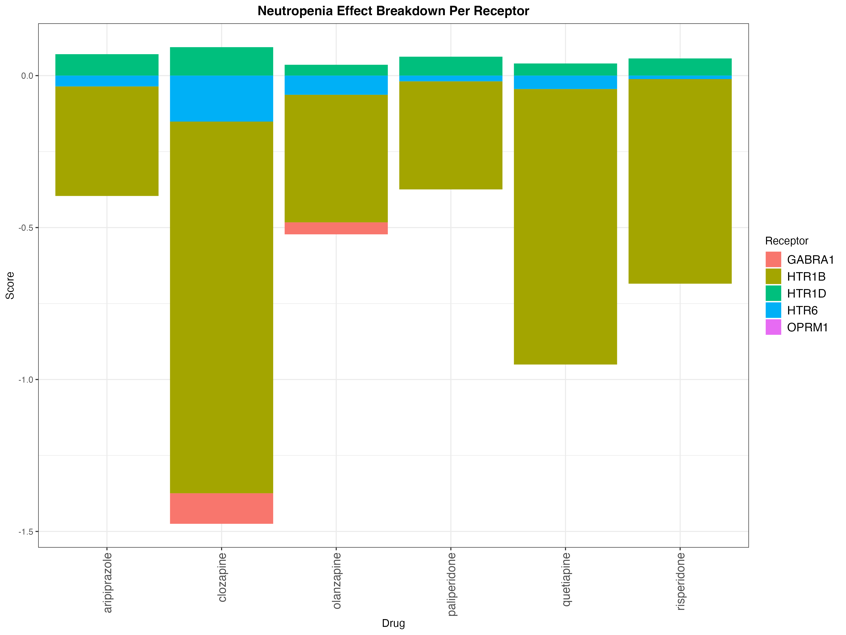 | 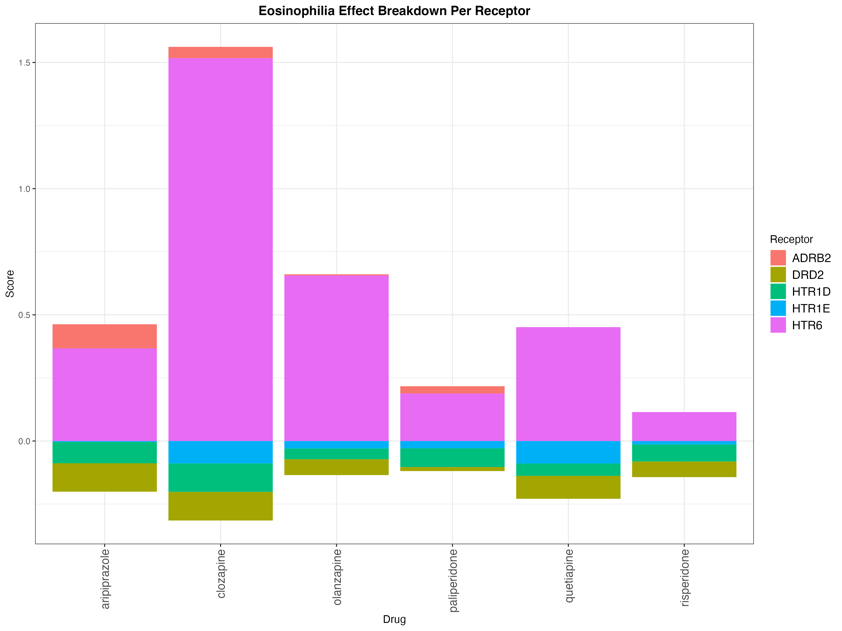 |
| --- | --- |
| 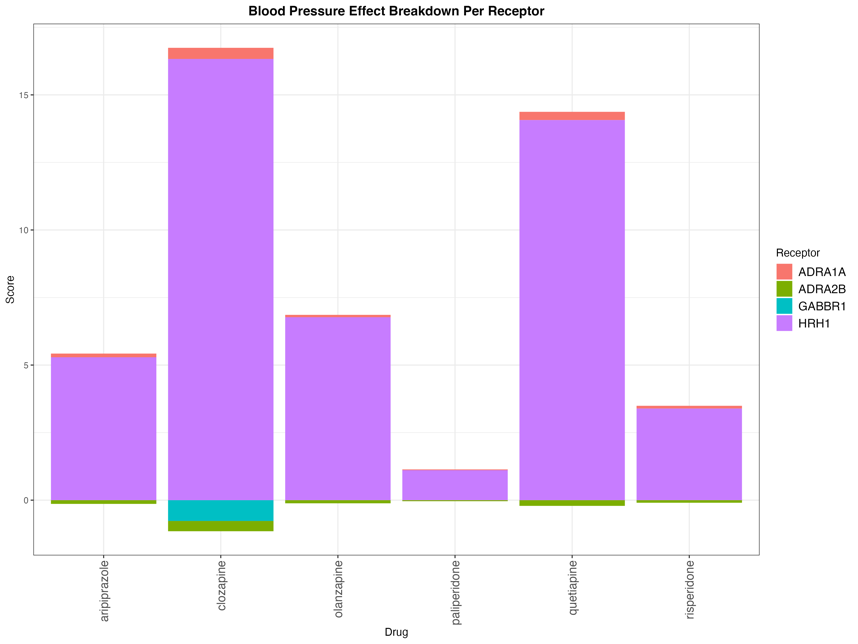 | 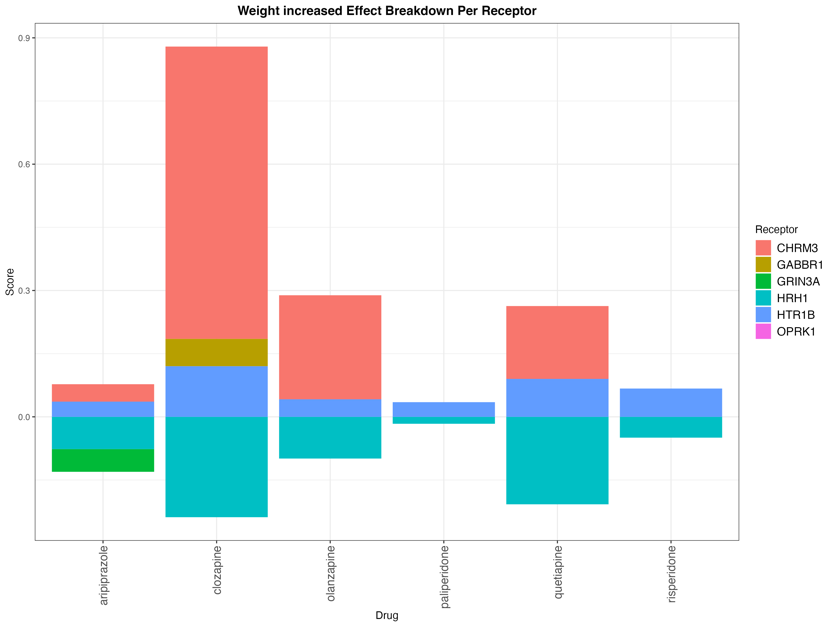 |
| 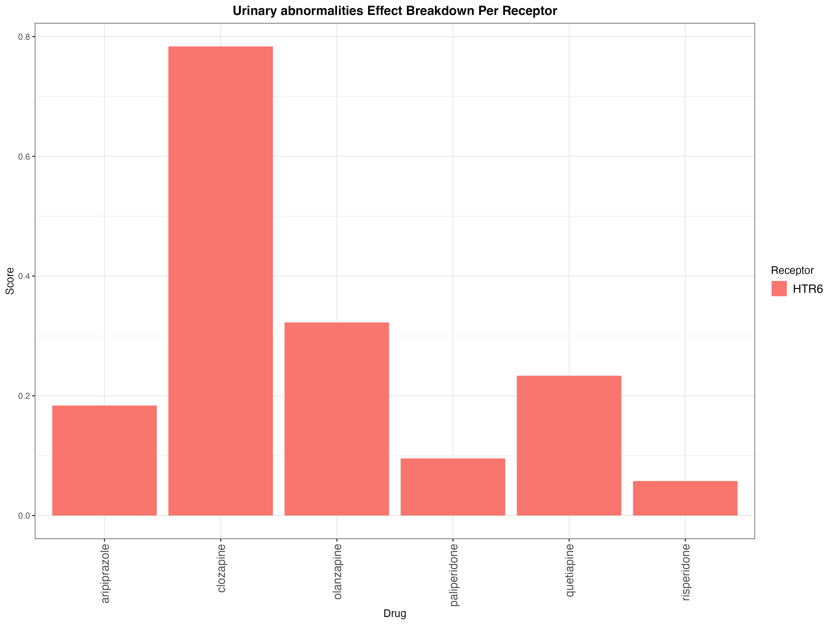 | 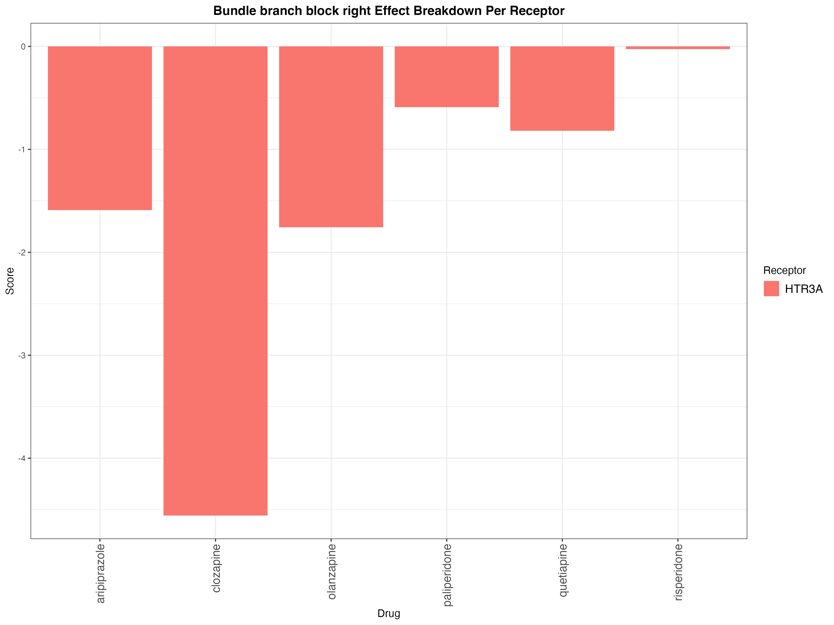 |
| 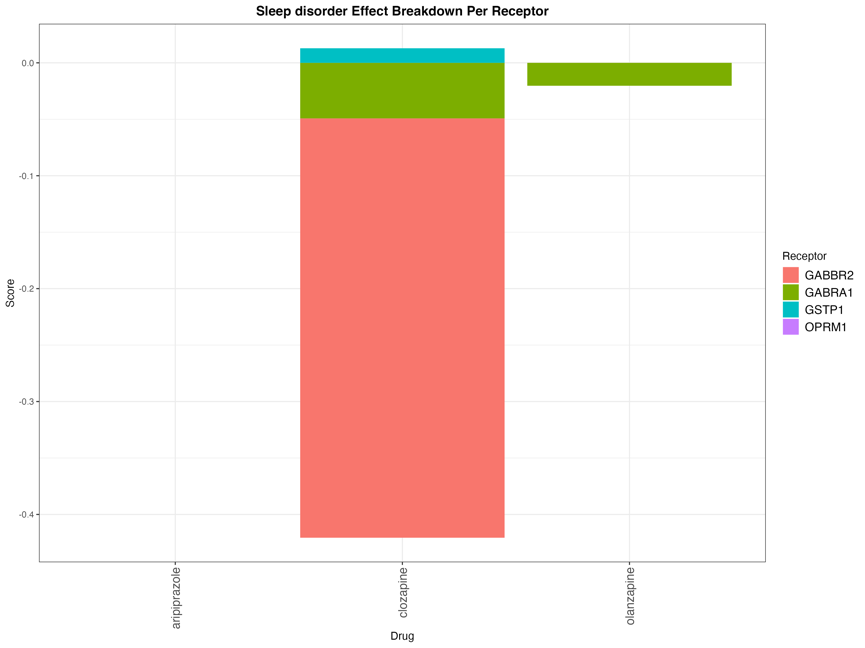 | 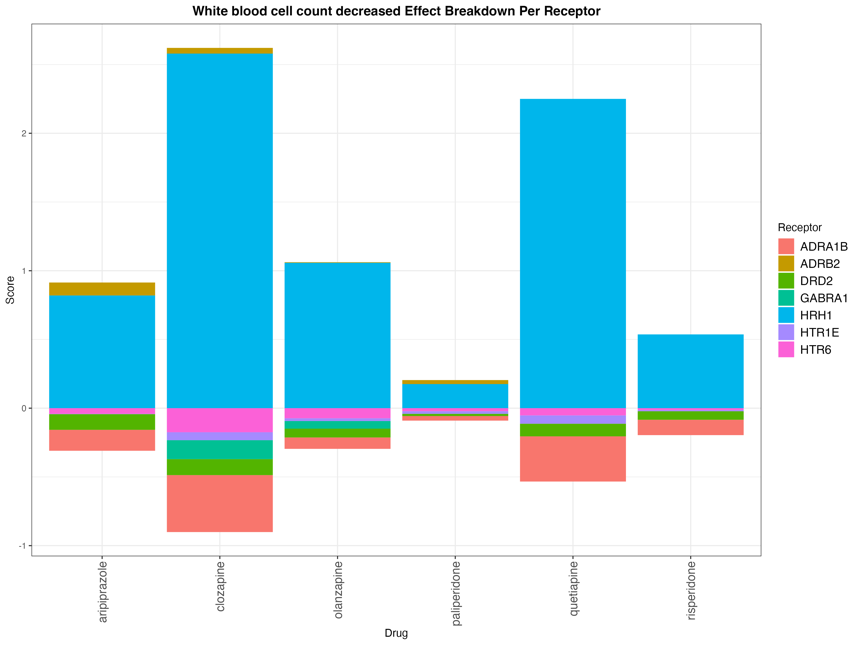 |
| 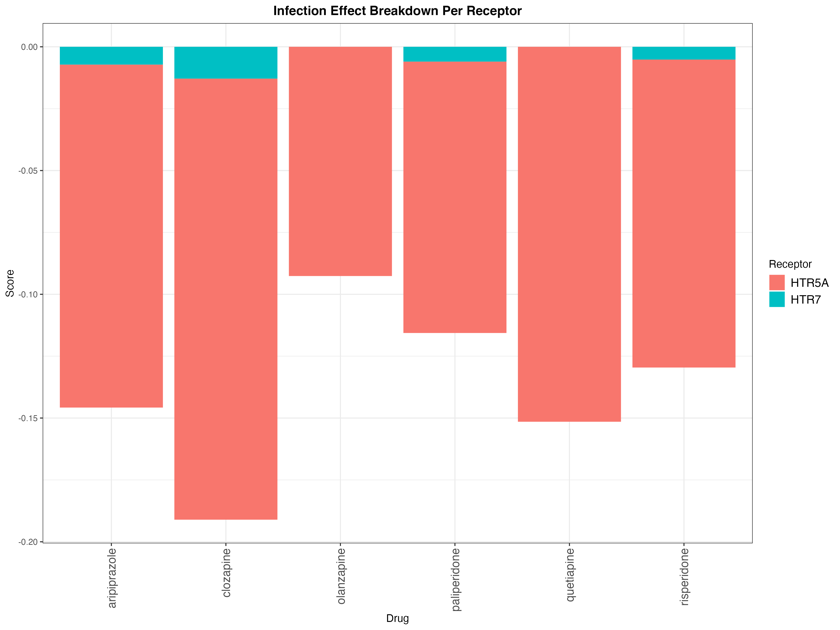 | 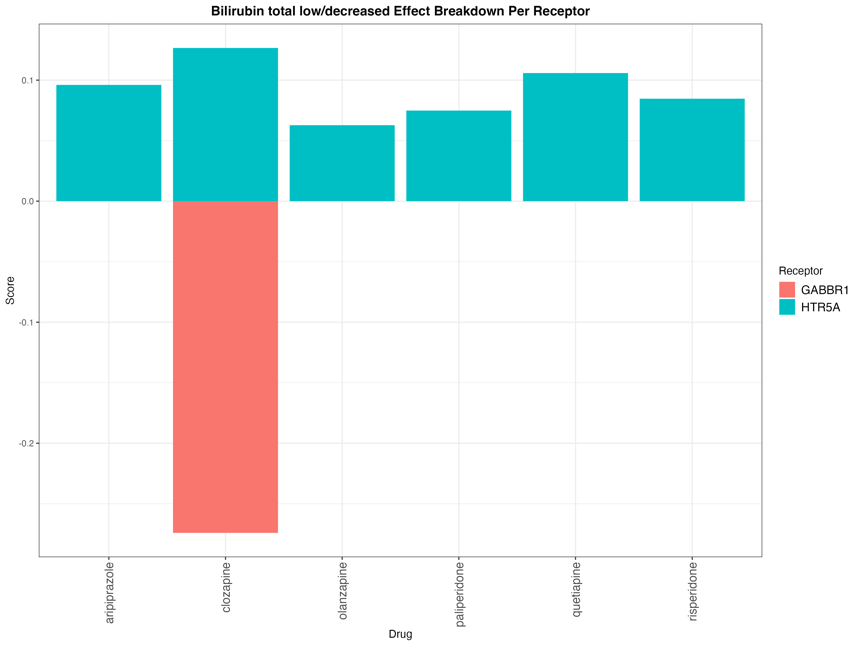 |
| 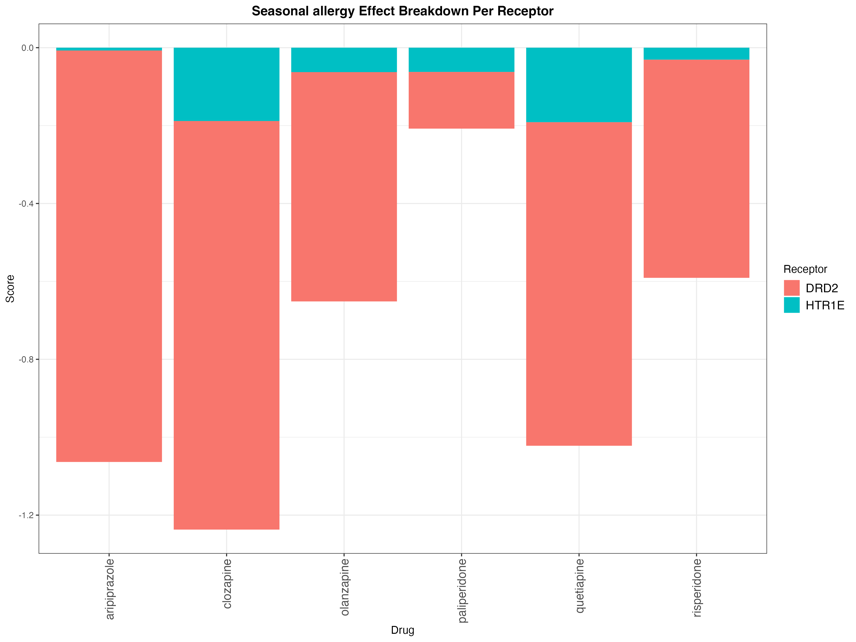 | 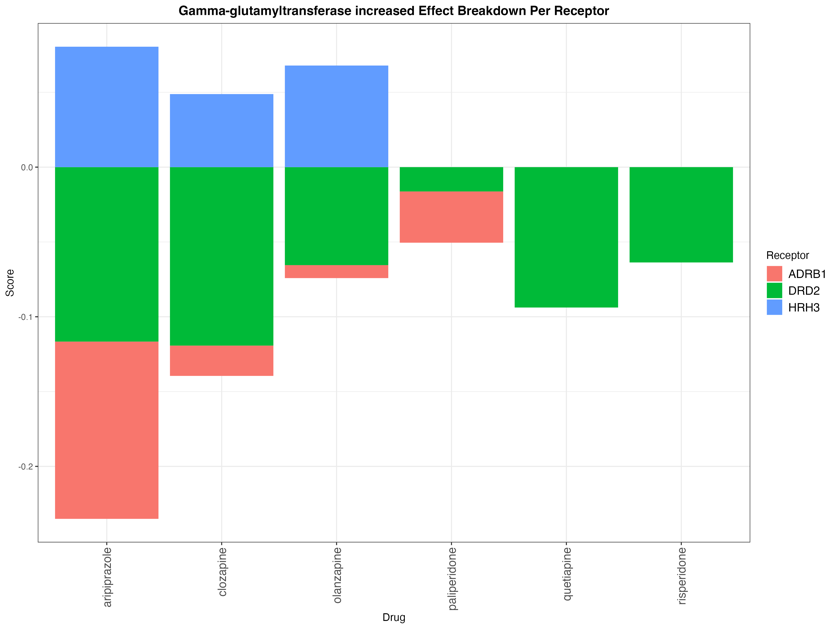 |
| 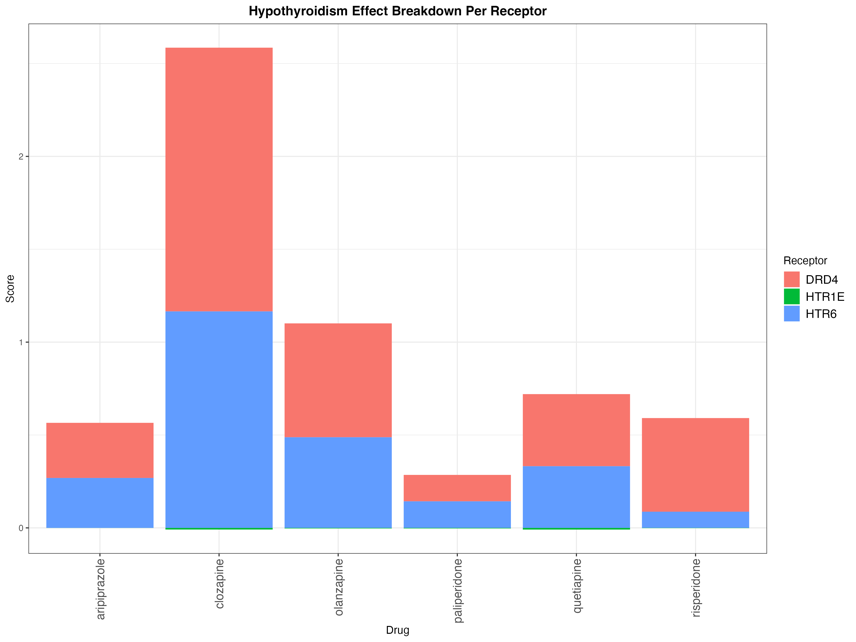 | 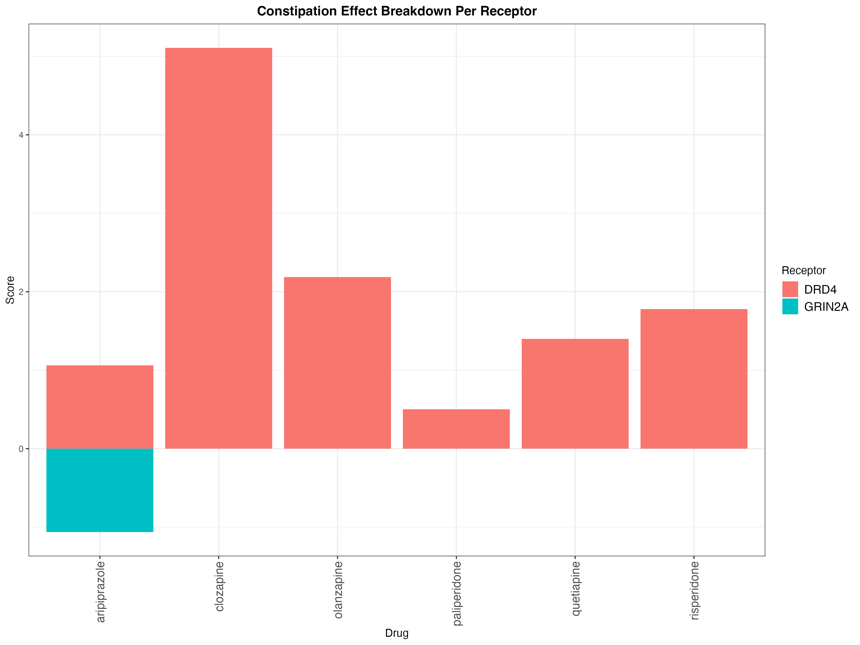 |
| 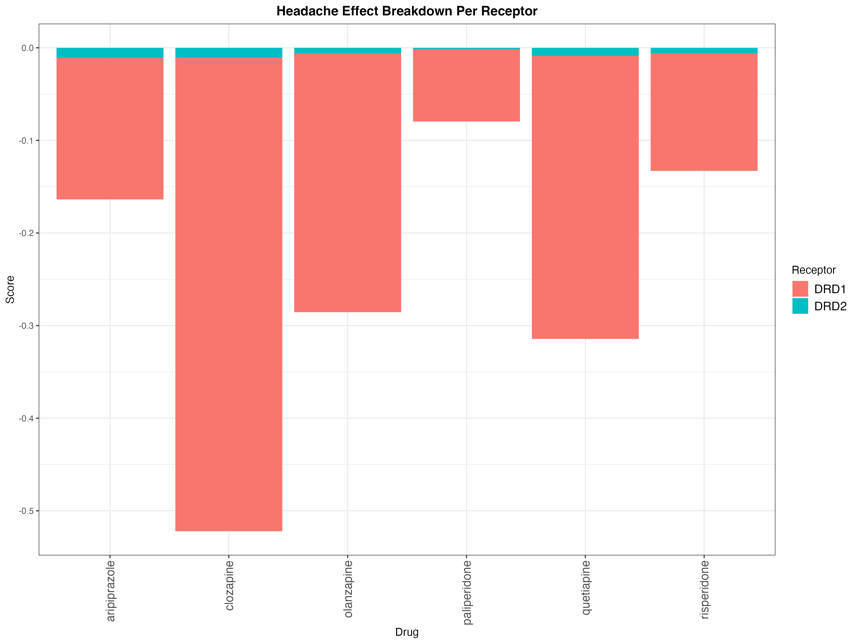 | 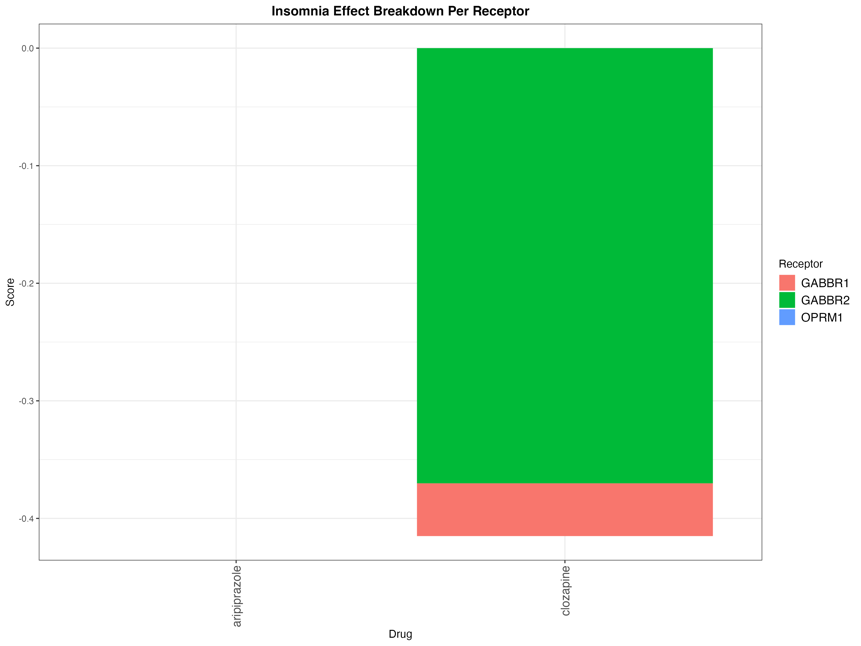 |
| 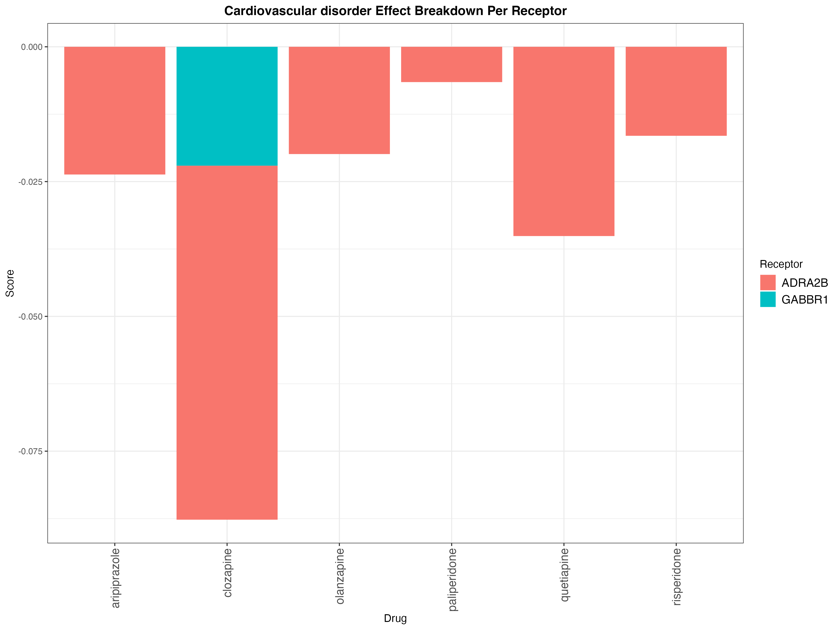 |  |

## Figure 2: Cumulative side-effects per drug across receptors

| 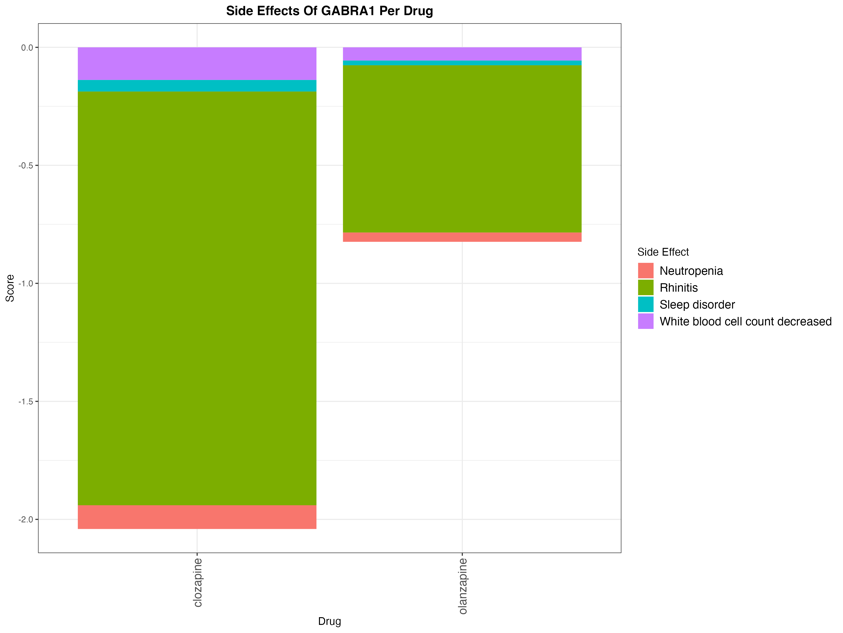 | 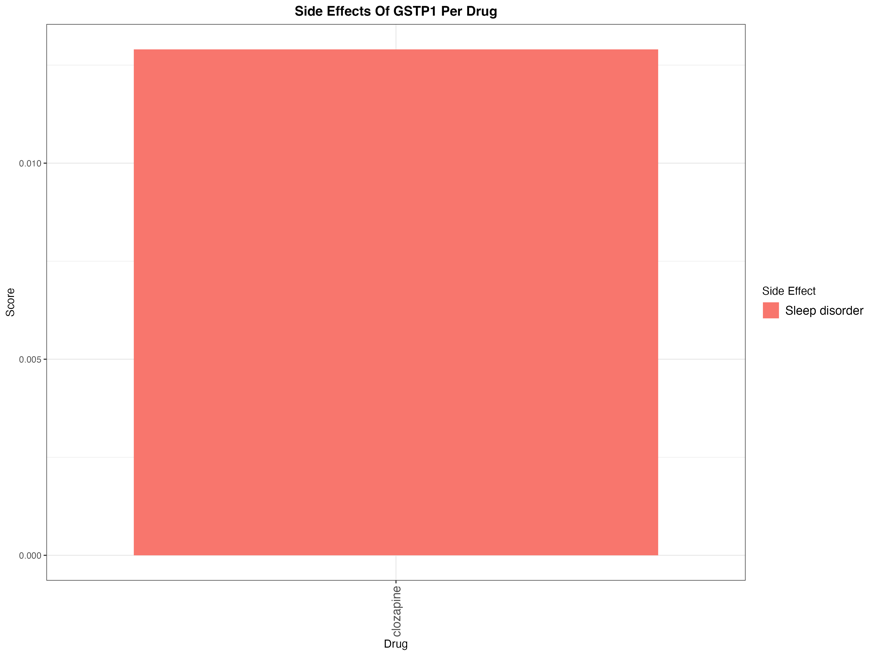 |
| --- | --- |
| 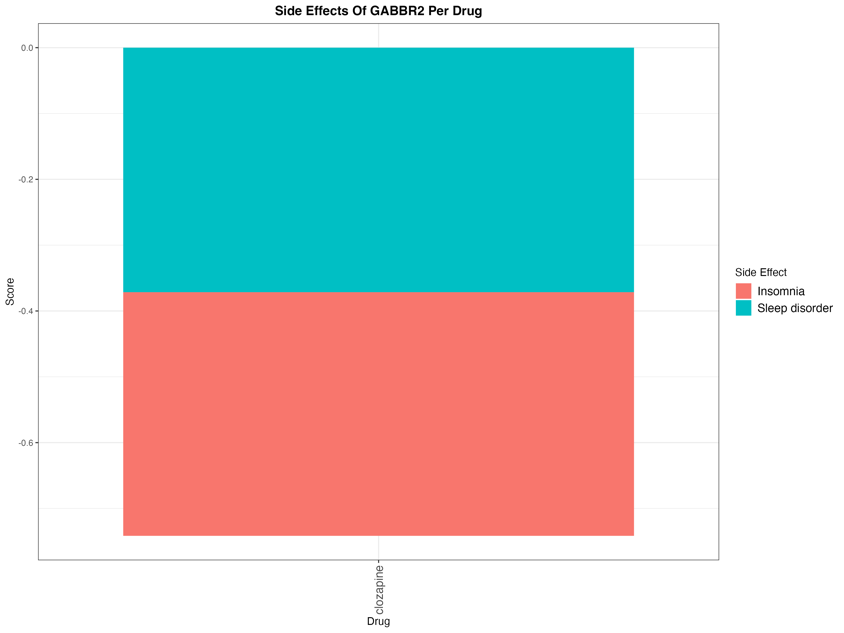 | 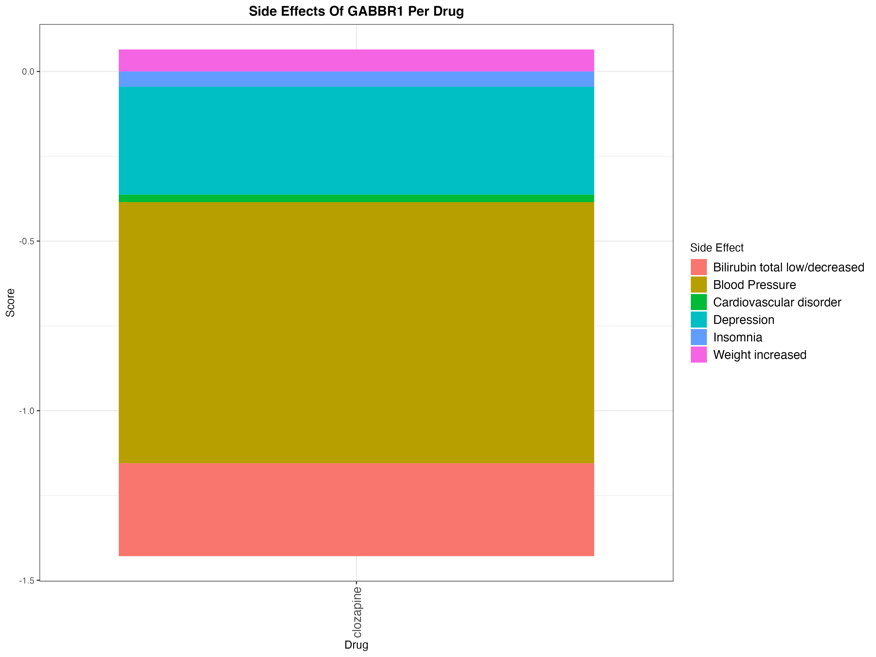 |
| 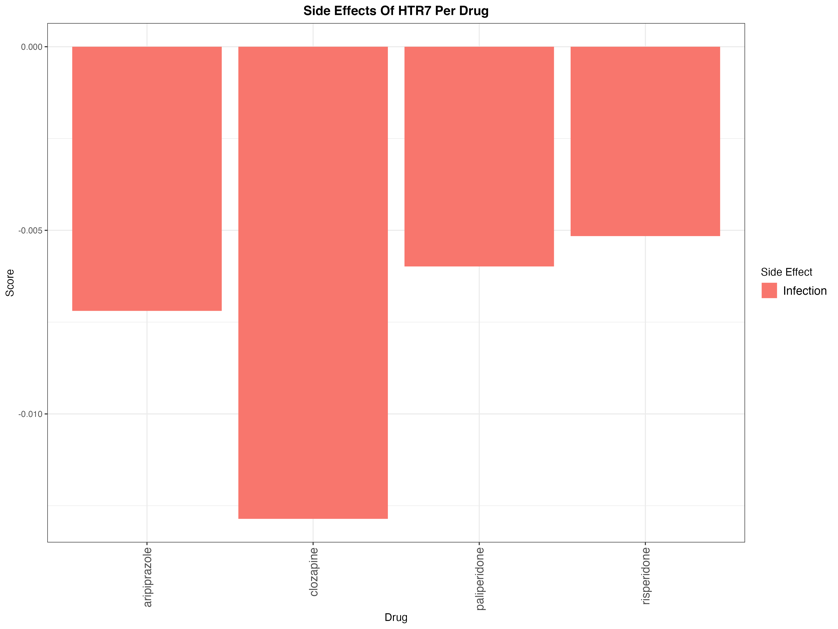 | 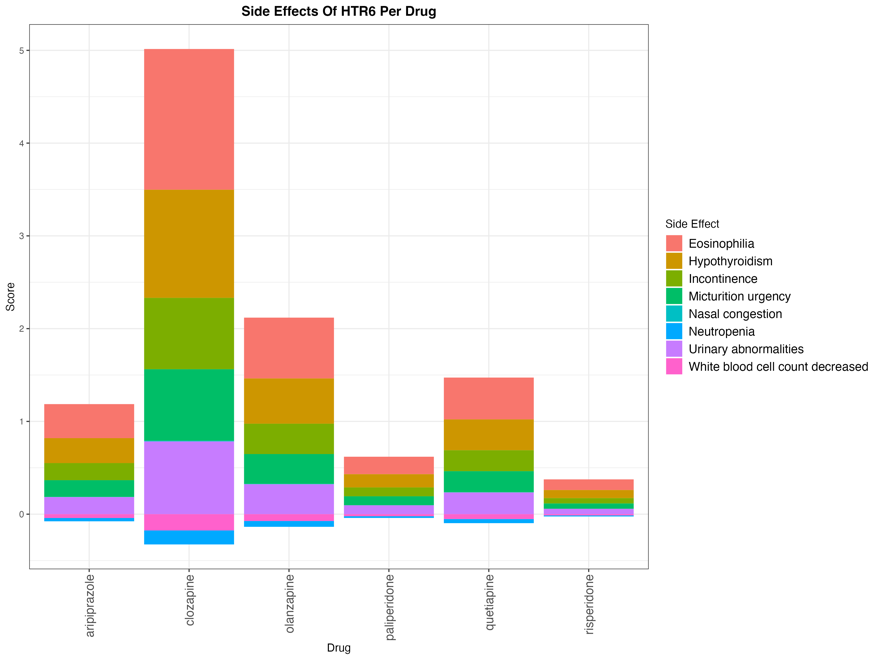 |
| 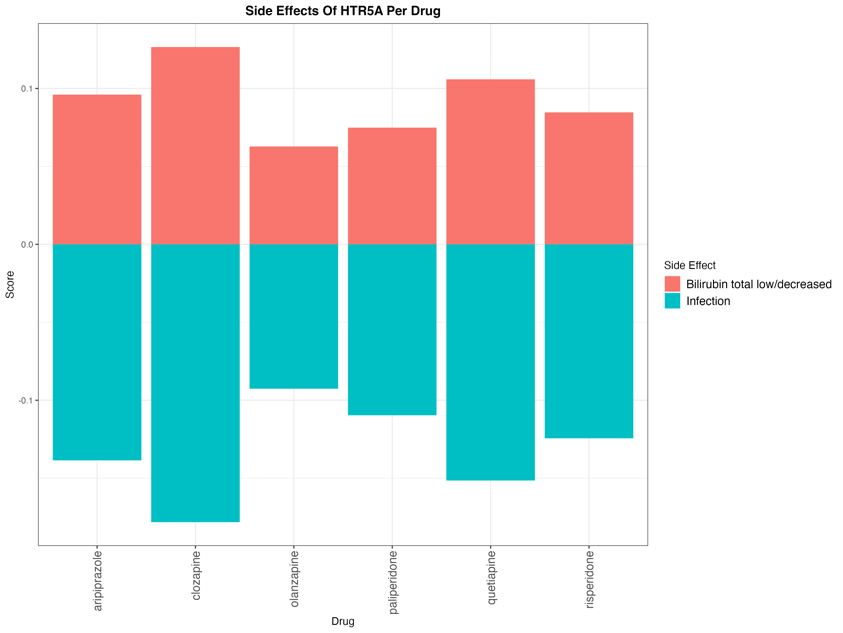 | 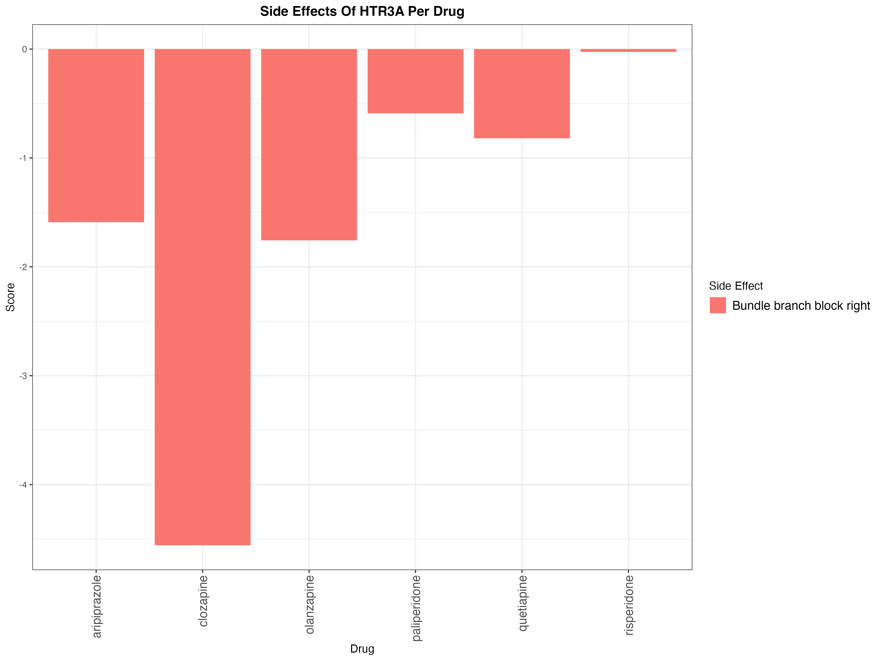 |
| 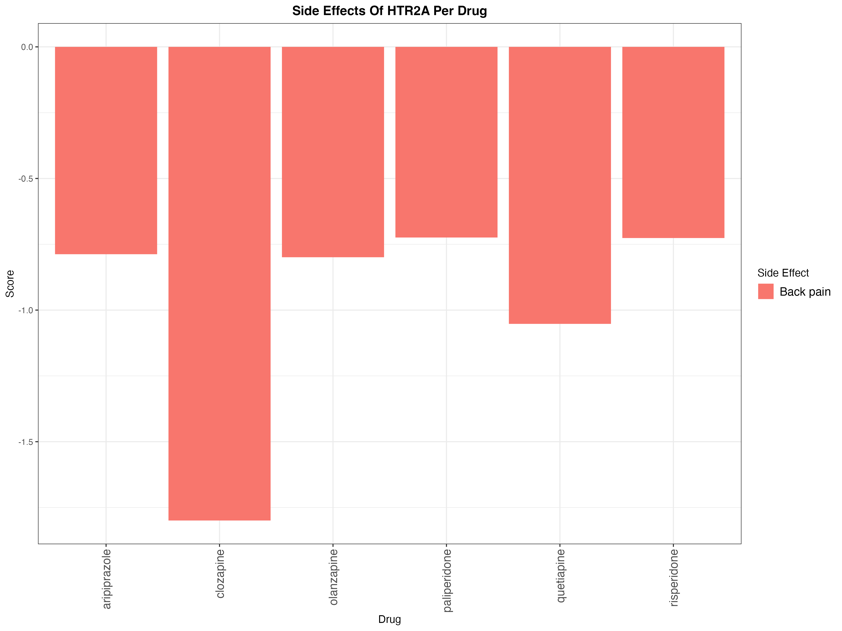 | 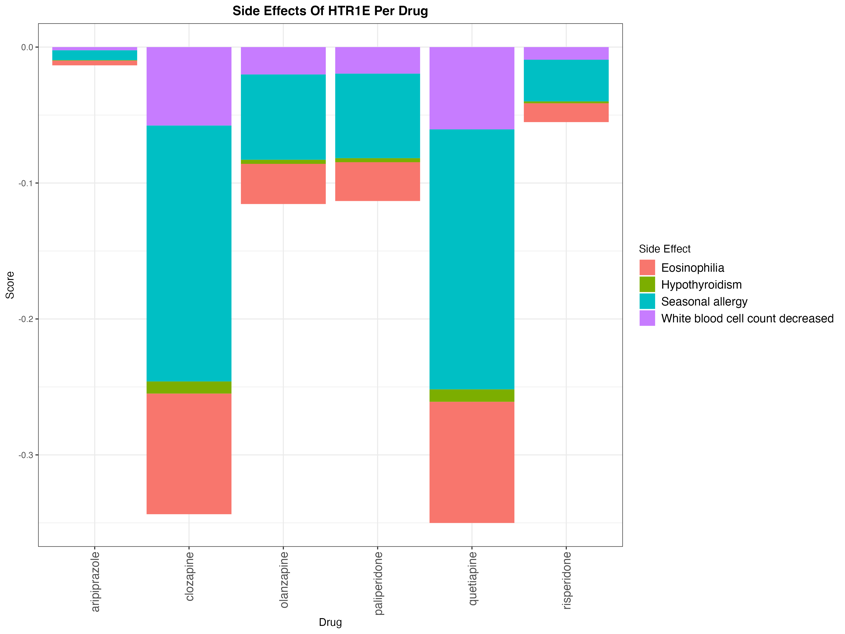 |
| 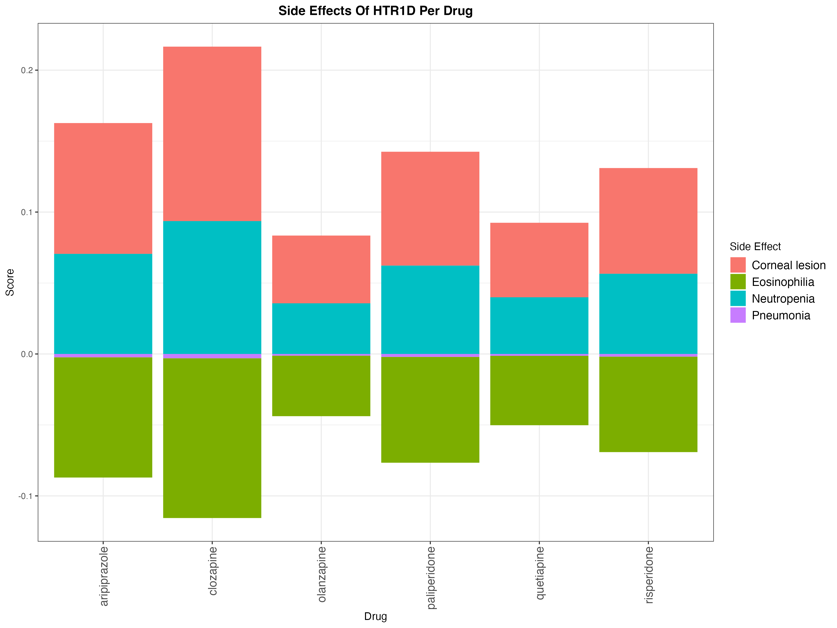 | 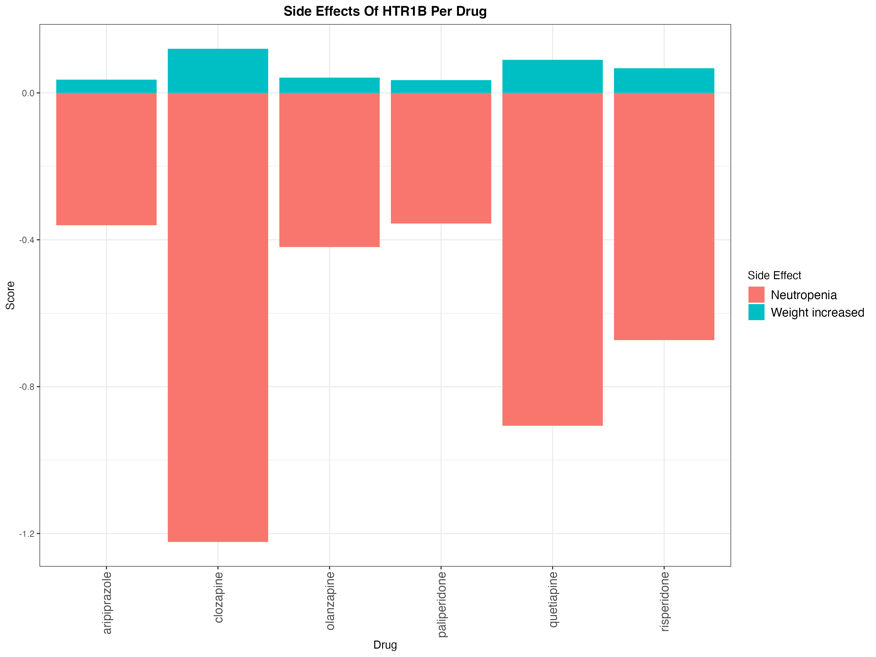 |
| 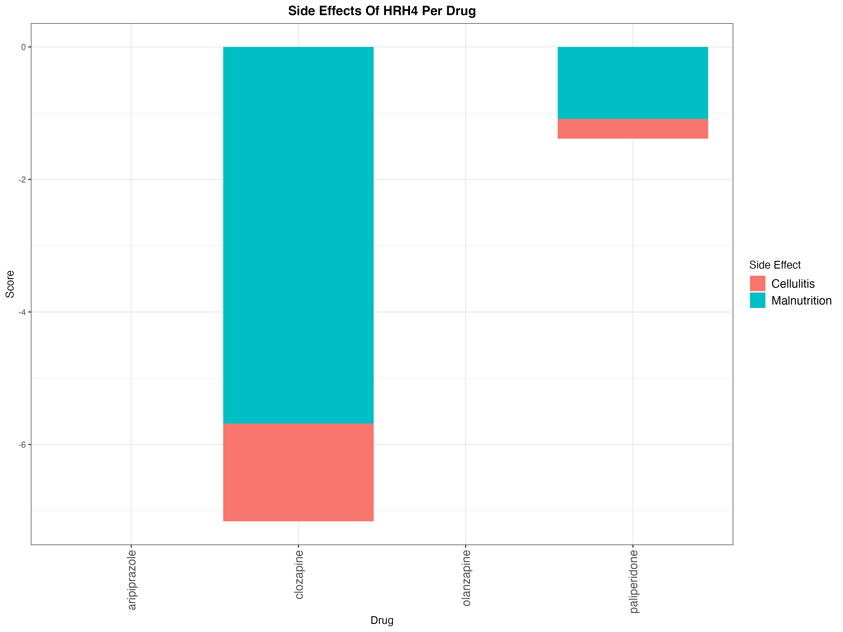 | 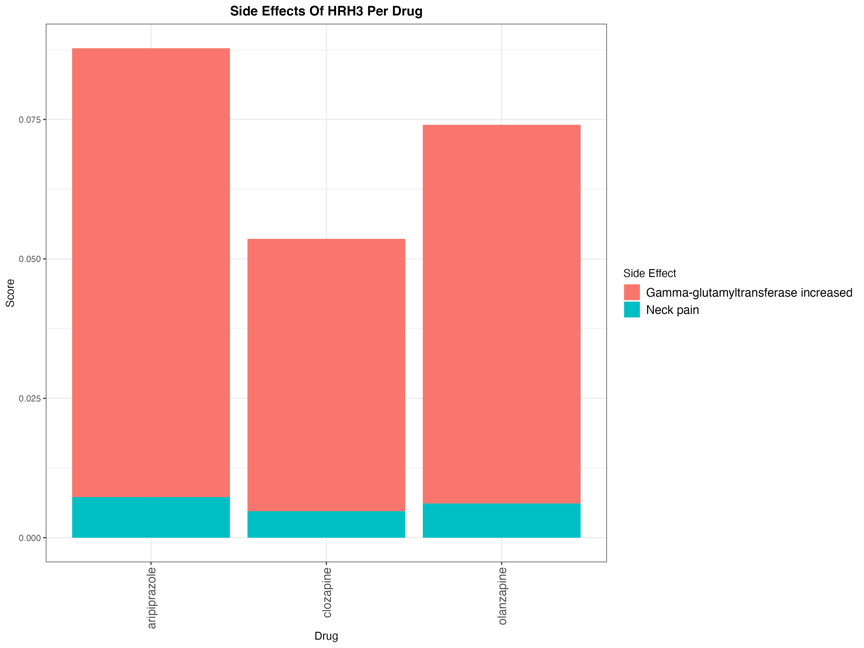 |
| 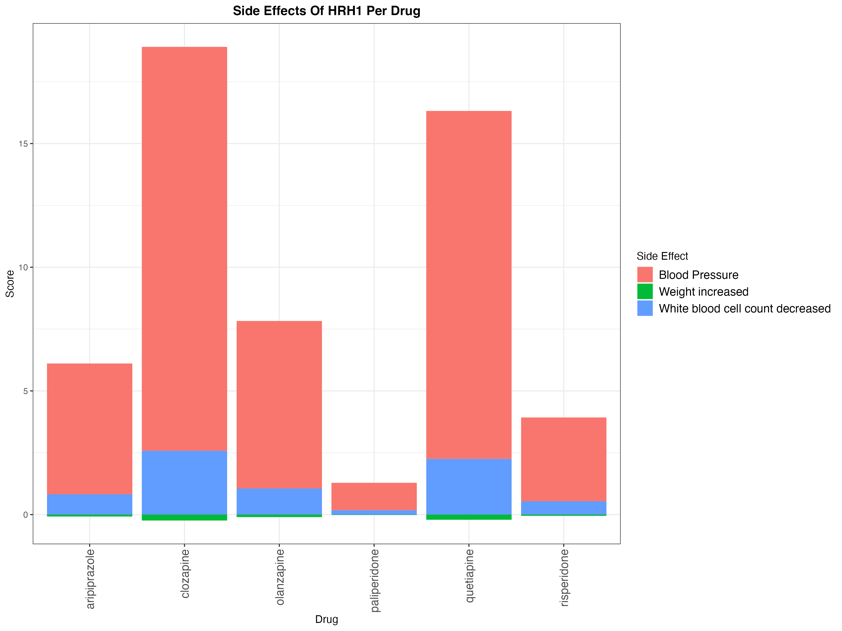 | 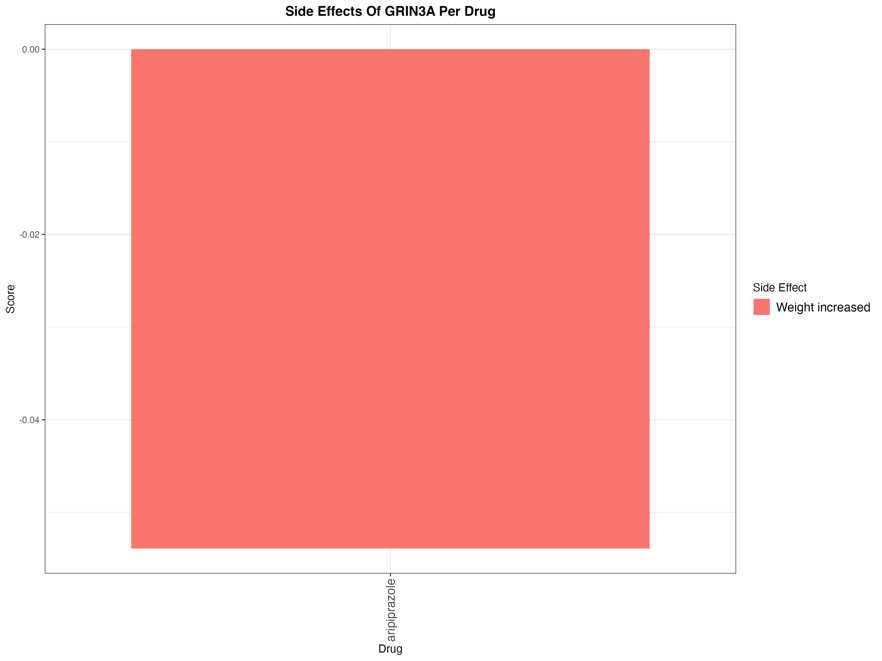 |
| 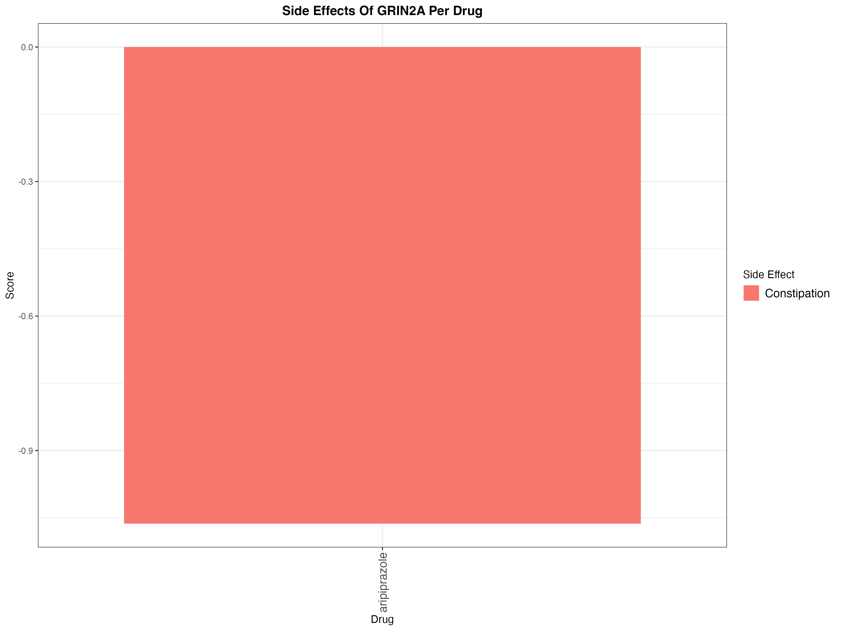 | 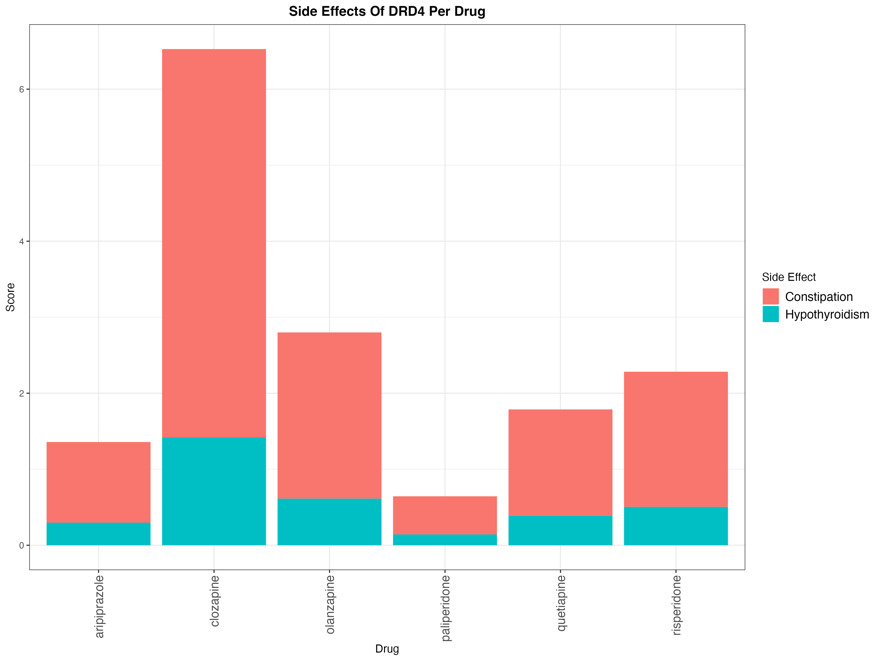 |
| 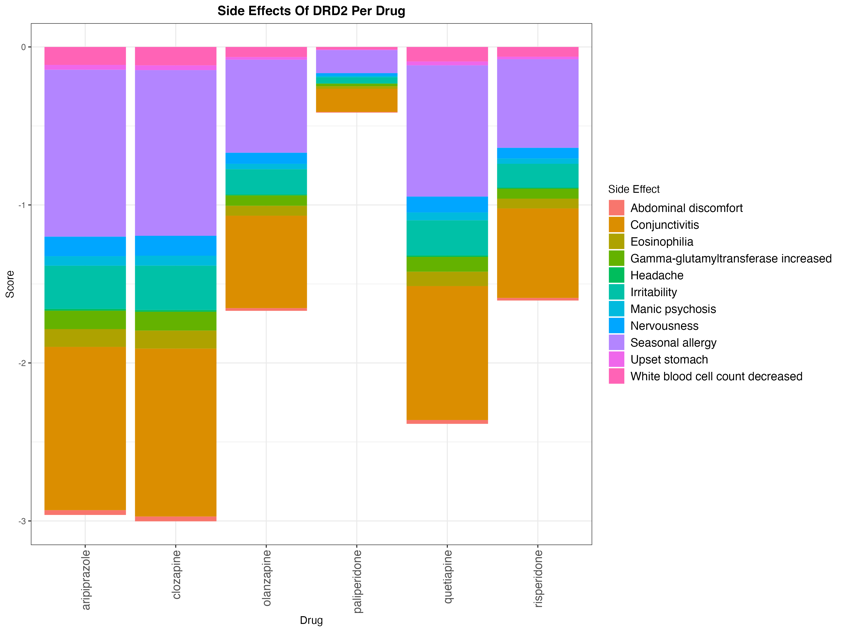 | 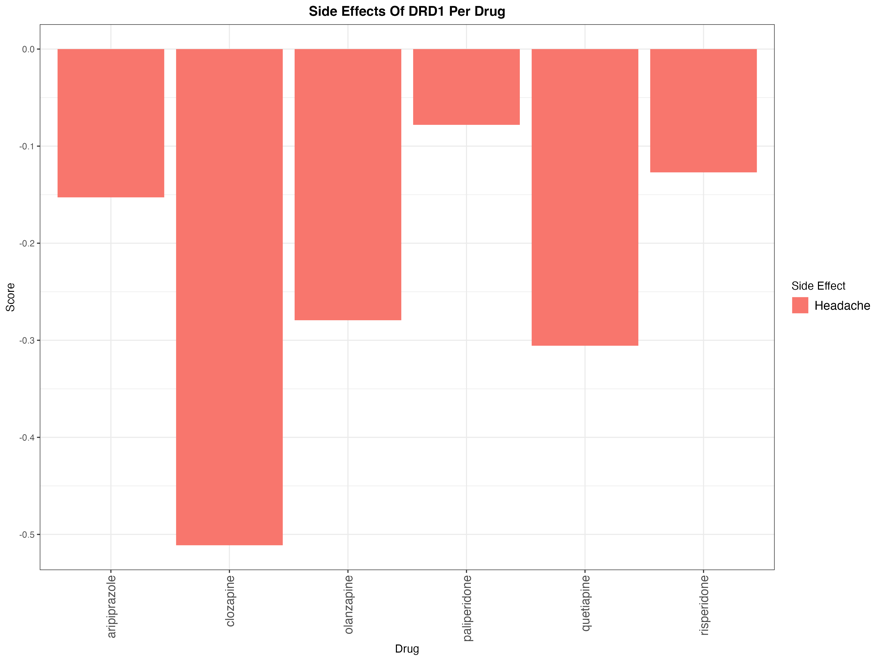 |
| 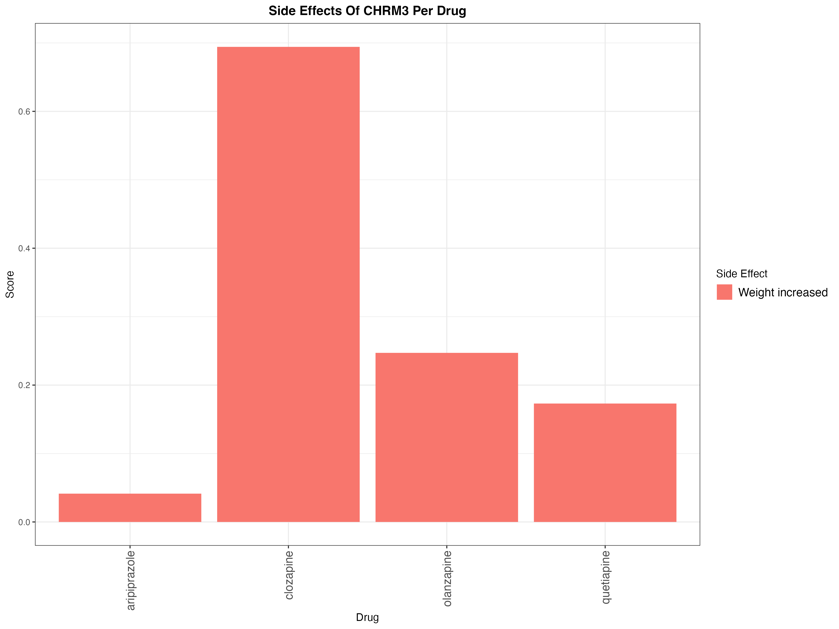 | 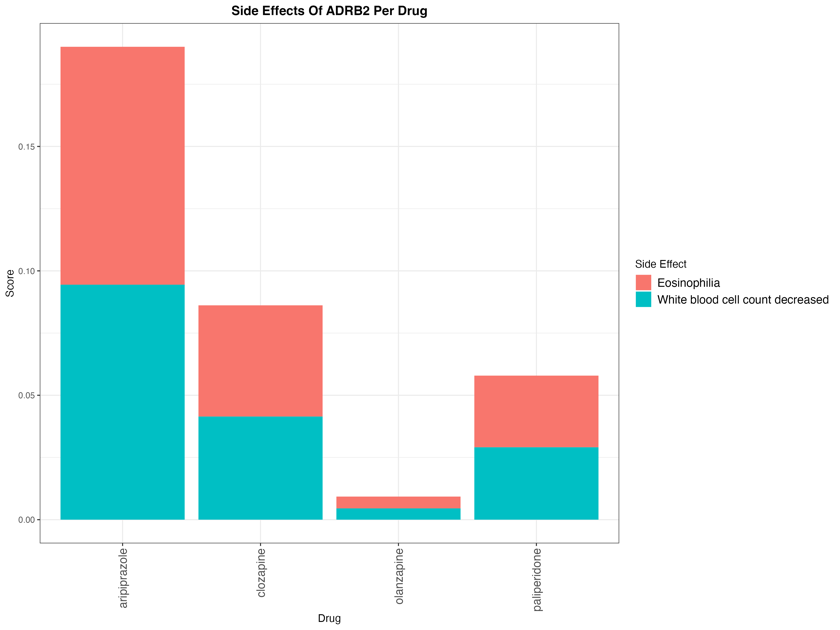 |
| 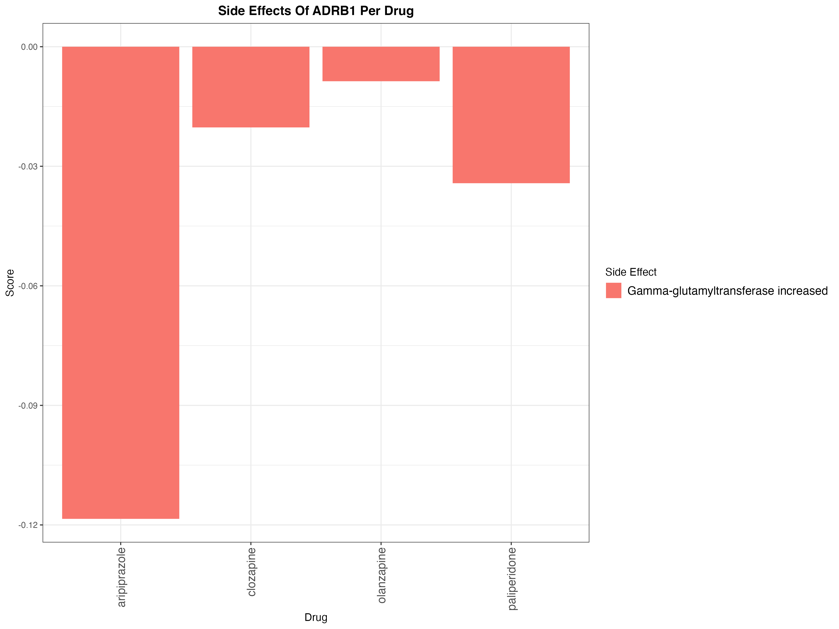 | 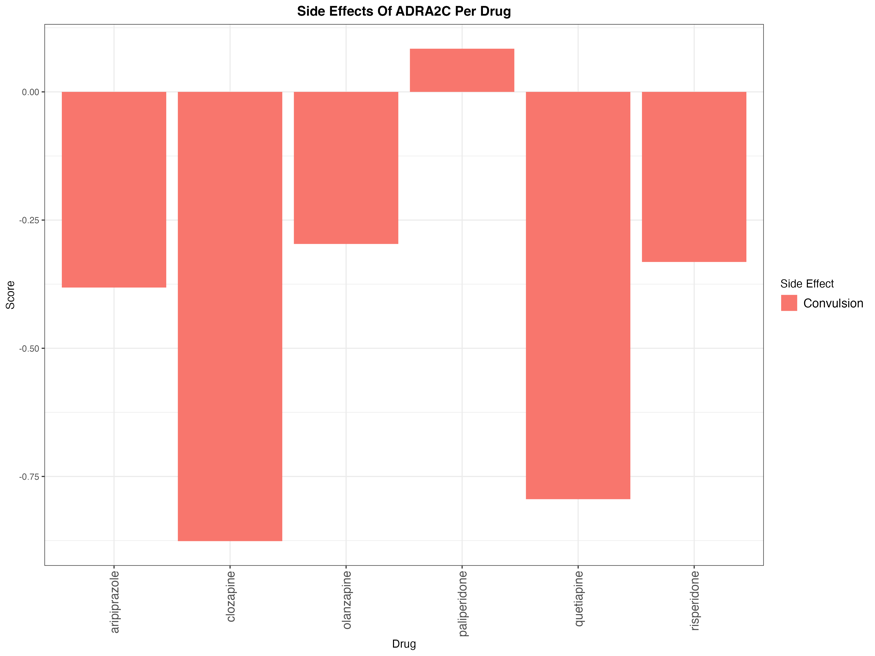 |
| 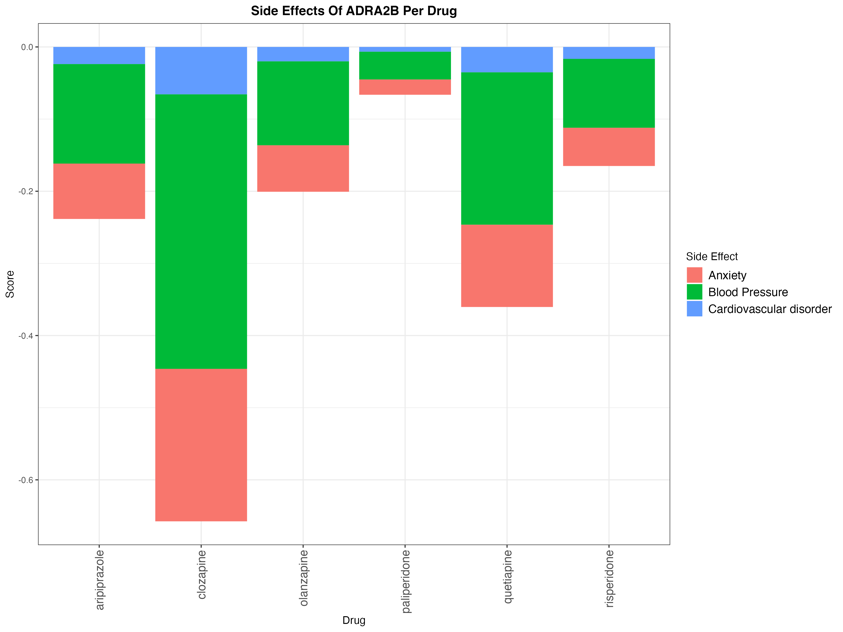 | 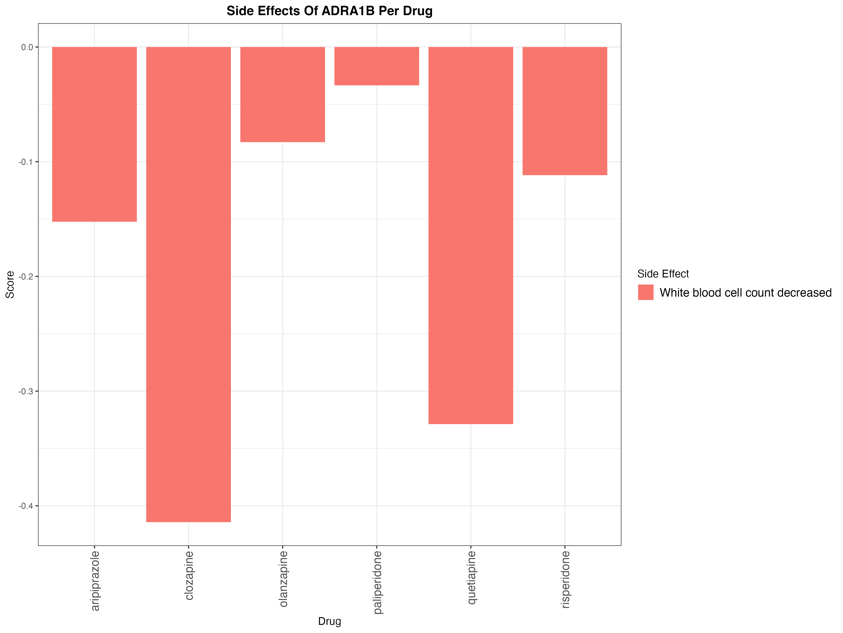 |
| 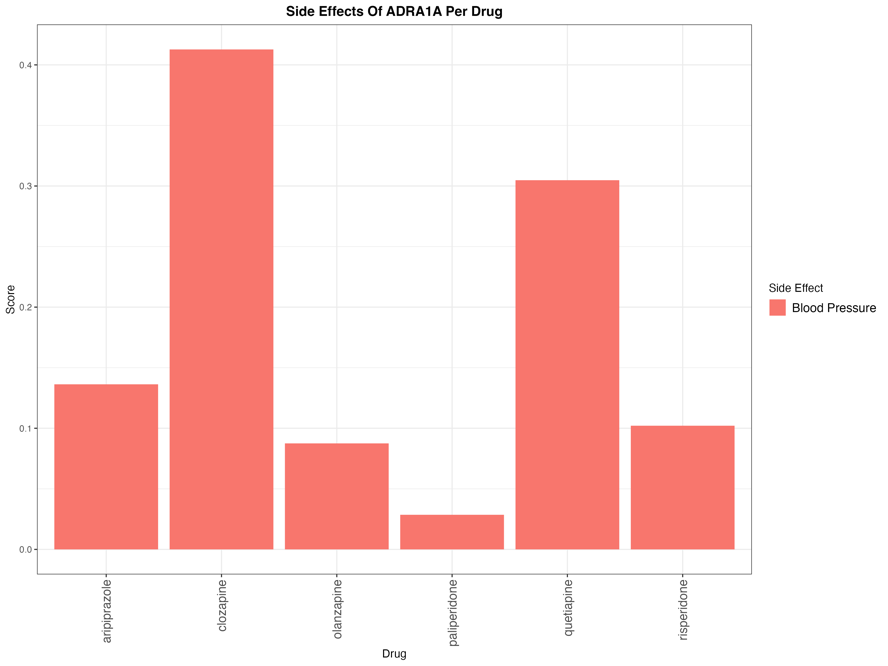 |  |

## Figure 3: Number of side-effects by receptor


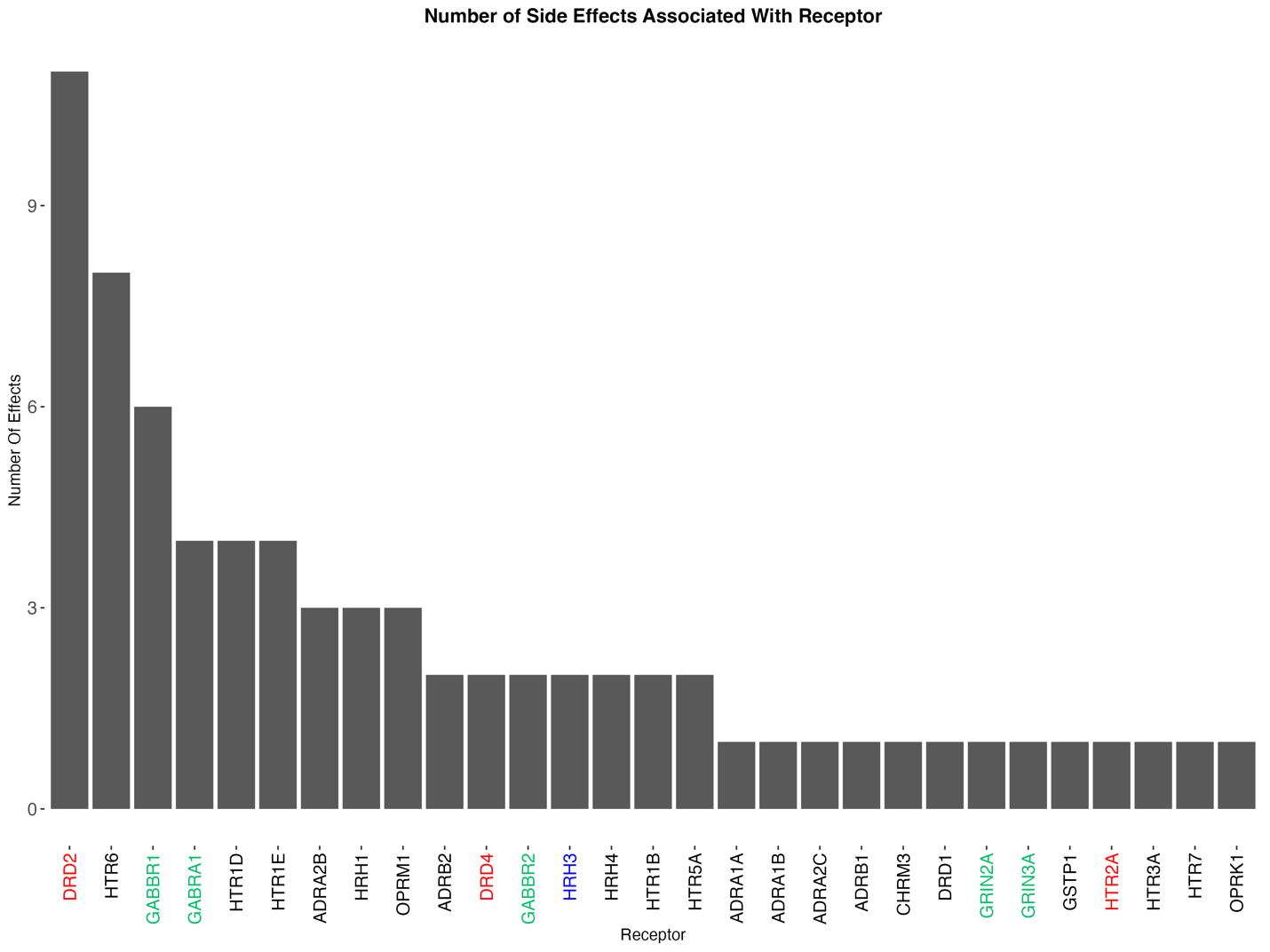


## Text 1: Explanation of Colocalization Thresholds

Typical colocalization analyses use a default threshold of H4 >= 0.8. However, in this analysis we are using more relaxed thresholds, where colocalization results required evidence of a shared effect to be H4 > 0.6, whilst also requiring evidence for distinct causal effects H3 < 0.1.

Unlike other hypothesis free investigations using GWAS summary statistics, we had a more specific hypothesis of 68 receptors we were investigating. As this investigation focuses on a smaller subset of SNPs, multiple testing correction is lower than the standard genome wide significance threshold. Colocalization is sensitive to strength of the signal it is analysing, which is shown in the original colocalization paper by [Giambartolomei](https://pubmed.ncbi.nlm.nih.gov/?term=%22Giambartolomei%20C%22%5BAuthor%5D) et al(1)., particularly Figure 3 and the surrounding discussion. The distribution of PP4 values vary with power.

Lowering the H4 threshold allows the differentiation between colocalization results that have the same causal effects but are less powered (H4), and colocalization results that have some evidence of different causal effects (H3).

Below is a linear regression of included colocalization results (where H4 > 0.6 and H3 < 0.1) where H4 is the response, and the p values are predictors. PheWAS p-value is a significant predictor with a negative slope on H4 (p-value: 3.84e-07), where the very small estimate values are indicative of the small scale of the PheWAS p-values (which range from 1e-5 to 7e-24). This relationship indicates a significant prediction that H4 will decrease as the lowest p-value in the summary stats increases.

1. > coloc_results <- coloc_results |> dplyr::filter(h4 > 0.6 & h3 < 0.1)

2. > lm(h4 ~ smallest_p_phewas + smallest_p_qtl, data=coloc_results) |> summary()

3.

4. Call:

5. lm(formula = h4 ~ smallest_p_phewas + smallest_p_qtl, data = coloc_results)

6.

7. Residuals:

8. Min 1Q Median 3Q Max

9. -0.23129 -0.06829 0.02497 0.06812 0.27690

10.

11. Coefficients:

12. Estimate Std. Error t value Pr(>|t|)

13. (Intercept) 0.85642 0.01186 72.226 < 2e-16 ***

14. smallest_p_phewas -988.65746 180.86573 -5.466 3.84e-07 ***

15. smallest_p_qtl -612.29868 255.88352 -2.393 0.0187 *

16. ---

17. Signif. codes: 0 ‘***’ 0.001 ‘**’ 0.01 ‘*’ 0.05 ‘.’ 0.1 ‘ ’ 1

18.

19. Residual standard error: 0.09704 on 93 degrees of freedom

20. Multiple R-squared: 0.2613, Adjusted R-squared: 0.2454

21. F-statistic: 16.44 on 2 and 93 DF, p-value: 7.674e-07

## References:

1. Giambartolomei C, Vukcevic D, Schadt EE, Franke L, Hingorani AD, Wallace C, et al. Bayesian Test for Colocalisation between Pairs of Genetic Association Studies Using Summary Statistics. PLoS Genet. 2014 May 15;10(5):e1004383.
