# Supplementary material for: Genetic inference of on-target and off-target side-effects of antipsychotic medications
Source: PLoS Genet. 2025 Jul 28;21(7):e1011793. doi: 10.1371/journal.pgen.1011793 (PMC12321077; doi:10.1371/journal.pgen.1011793)
Supplement: Attachment — Submitted filename: Response to Reviewers.docx [file pgen.1011793.s005.docx]

# Response to Reviewers

**Author response:** We thank the reviewers for the careful evaluation of the manuscript. The comments were very helpful and constructive, and we have taken all the comments into consideration for this resubmission. Below, we have a point-by-point response to each of the reviewer comments, and the corresponding changes are indicated in the manuscript as indented red text.

## Reviewer 1:

We would like to thank reviewer 1 for their time and effort in reviewing our manuscript. We have responded to individual points inline.

**Although in their conclusions the authors claim that this approach could be used to help inform drug choice for patients and clinicians, perhaps they need to specify the mechanisms of the clinical utility of their approach, especially in light of the stringent requiring of pharmacogenomics implementation.**

Thank you for the comment. Instead of intending to provide direct recommendations to clinicians, we are providing our new evidence as a means to investigate drug repurposing. We felt it was outside the scope of the paper to investigate pharmacogenomic implementation. Therefore, the results provided are not immediately translatable but could be used in a translational context in the future. We have updated the manuscript to further clarify this with the following text:

The results shown and subsequent discussion regarding the possibility of drug repurposing are not intended to be taken as clinical guidance for antipsychotic use. Rather this work is intended to illustrate potential value of our approach to understand mechanisms of drug side effect. We are providing a novel method which provides insights into the mechanisms of drugs which may lead to future pharmacogenomic investigations. The results provided are not immediately translatable for informing personalised drug choices for patients.

**I was tried to identify some sort of “a positive control” (a drug with a known side effect(s) that is mediated by a known genetic factor(s)” for the approach used by the authors. If they did, then the authors might want to clarify this.**

Thank you for your suggestion of a “positive control”. We have also attempted to identify a positive control for this study. However, the difficulty lies in the fact that existing side-effect literature is unreliable due to known reasons, namely the lack of large-scale random control trails for medication, and inconsistent data collection and reporting.

Adverse event databases like SIDER (which we use), and the type of literature review that is provided by Stroup et. al(1) whom provide the less precise “+” categorical groupings are currently some of the best resources for known side effect profiles.

The type of evidence that we can reference with existing literature is the same evidence that we have provided in the ‘existing literature’ column in *Table 2: Table of Highlighted Side-effects* for those specific receptor / side-effect pairings.

Investigating the presence or absence of a side effect remains difficult, as many of these drugs are targeting similar receptors, which therefore manifests in a significant overlap of side-effects. We’re using the receptors as instruments to predict side-effects, as opposed to using this in a pharmacogenetics context.

**Comments on the potential utility of this approach for drugs used for the treatment of other common conditions such metabolic conditions and cancer might be useful for the readers.**

Thank you for the suggestion. We have expanded the discussion section to elaborate on how this approach can be used for other drugs and conditions.

Further, this approach could be used to investigate any drug that targets a wide range of receptors and has a wide range of reported side-effects, leading to novel insights across a range of diseases.

## Reviewer 2:

We would like to thank reviewer 2 for their time and effort in reviewing our manuscript. We have responded to individual points inline.

**1. The effect score of side effect per receptor is well-defined but lacks evaluation. Table 1 uses subjective "+" symbols to compare scores with existing literature, lacking quantitative alignment (e.g., statistical tests, correlation coefficients).**

**This undermines the credibility of claimed corroborations. I believe a more systemically quantitative instead of qualitative evaluation will showcase the benefits of such definition of effect score. For example, scores can be compared to real-world side effect incidence (e.g., clinical trial data or adverse event databases).**

Thanks for your comment. This is a pertinent point, as it highlights the current lack of robust quantitative literature for the side-effects of drugs. This lack of literature is one of the reasons that this type of investigation is necessary. Adverse event databases like SIDER (which we use), and the type of literature review that is provided by Stroup et. al(1) whom provide the less precise “+” categorical groupings are currently some of the best resources for known side effect profiles. Existing side-effect literature is unreliable due to known reasons, namely the lack of randomised control trails for medication, and inconsistent data collection and reporting.

Side-effect data collection occurs in two different axes of investigation; one is frequency and the other is severity. As an example, Stroup et al. combines the severity and frequency of side-effects and reports them together as a categorical variable (number of ‘+’s). The axis in which the side-effect is reported on is usually dependant on the side-effect. For example, neutropenia is a serious but rare side-effect, so severity is usually discussed.

Attempts to improve this comparison have proven to be difficult. We tried to compare the calculated scores to the upper and lower bounded reported frequencies of side-effects collected by SIDER, however there are many reasons why this is not sufficient: significant amounts of missing data, wide frequency ranges, and as previously discussed, frequency ranges alone are not a sufficient metric to compare our side-effect scores against.

The type of evidence that we can reference with existing literature is more specific gene / side-effect pairings, however this is the same evidence that we have already provided in the ‘existing literature’ column in *Table 2: Table of Highlighted Side-effects* for the gene / side-effect pairings of interest.

We have attempted to be clear about the limitations of our quantitative prediction, namely outlining all known possible issues in Figure 2 of the manuscript. We have added additional text in the ‘Limitations’ section of ‘Discussion’ highlighting the lack of existing quantitative metrics in which we can meaningfully compare our results with.

**2. The study would benefit from using pQTLs over eQTLs in MR due to the following reasons:
• pQTLs directly reflect genetic regulation of protein levels, which are more proximal to drug-receptor interactions and downstream phenotypic effects (e.g., side effects). Drugs act on proteins, not mRNA. Using pQTLs aligns with the pharmacological mechanism of antipsychotics (e.g., receptor binding affinity).
• eQTLs measure mRNA expression, which does not always correlate with protein abundance due to post-transcriptional regulation (e.g., translation efficiency, protein degradation). The authors themselves note that eQTL and pQTL directions disagree in ~30% of cases, undermining confidence in eQTL-based causal estimates.**

Thank you for your response. We agree that pQTLs would be preferred due to the reasons you provide. However, only 2 out of the 68 genes of interest exist in the UK Biobank Pharma Proteomics Project (UKB-PPP). Those two receptors are GSTP1 and SMPD1. Due to a lack of overlap between the receptors of interest and available pQTL measurements, we decided to focus on eQTLs. We have updated the manuscript to include this shortcoming in the limitations section.

Although pQTL measurements would likely be a better proxy for receptor binding of a gene, data are currently lacking. Only 2 out of the 68 genes of interest exist in the UK Biobank Pharma Proteomics Project (UKB-PPP).

**Moreover, the study relies on brain-derived eQTLs (MetaBrain) for systemic side effects (e.g., neutropenia, blood pressure), which is biologically implausible. For example, neutropenia (low white blood cells) likely involves receptors in hematopoietic tissues (e.g., bone marrow), not brain. Blood pressure regulation involves vascular or renal tissues.**

Thank you for your response and we agree with the difficulty of this limitation. We believe that the documented correlation of gene expression across tissues, via pleiotropic effects, allows us to still infer meaningful and interesting results. We did not rely solely on Brain QTLs, but data from two sources, MetaBrain QTLs and eQTLGen, to leverage blood gene expression data as well. However, we did still utilise results that presented themselves via tissues that may not be biologically relevant.

We relied on the documented phenomena that cis eQTLs tend to be correlated and colocalizing across different tissues(2). Combining this knowledge with the specific phenotype / gene pairing of the colocalization we are performing (which is determined via side-effects and drug targets) allows us to perform specific tests. Finding colocalisation in the cortex for Neutropenia is not evidence that it acts through the cortex, but the fact that it still colocalises highlights an effect, which is likely pleiotropic. Our method does not report which specific tissue the receptor linked to.

Ideally, we would look at every tissue that a side-effect might act through and run colocalizations on that. For some side-effects, the causal mechanism is simple or well understood (Neutropenia), but for others, the causal mechanism for every side-effect is not known, or are sufficiently complex (weight gain). Most side-effects are both polygenic and occur through more than a single causal pathway.

One alternative potential solution to ours would involve using most or all known tissue specific QTL datasets to provide more specific results, which would require a new order of magnitude of computational complexity that we are unable to undertake. Using Brain regions and blood eQTLs, we already have multiple colocalizations across different tissues. The difference in which tissue we ultimately used did not significantly change the results, as the computed Wald ratio size or direction of effect was not greatly altered.

This issue is similar to the issue that binding affinity data is similarly not sufficiently reported on in a tissue specific manner, which we have also highlighted as a limitation in the paper. We attempted to highlight the difficulty of relevant tissues in figure 2. We have expanded the point in the figure and in the methods section to highlight not only relevant tissues in drug interaction, but also in relevancy of eQTL tissue.

Figure 2 Legend:

**Relevant Tissue:**

Problem: Drugs do not only interact with one tissue in the body, they can affect different systems, of which a single QTL measurement may not capture, especially when comparing using genetic colocalization.

Resolution: Use different eQTL datasets to capture the different. We used both MetaBrain and eQTLGen. Rely on pleiotropic eQTL effects which correlate across different tissues.

**3. Interpretation of Binding Affinity and Drug Dosing. The study integrates binding affinity data (Ki values) into the score However, binding affinity alone does not dictate receptor activation. Some drugs act as partial agonists or inverse agonists, which could influence the interpretation of receptor-side-effect relationships.**

We agree with the idea that binding affinity alone doesn’t dictate receptor activation. We attempted to highlight the limitations of knowing which direction of effect the drug-receptor binding would have through many means and attempted to summarize those difficulties in figure 2. The binding type (ie. antagonist, agonist, partial agonist), coupled with the inability to a priori infer receptor-side-effect relationships. This led to a limitation in our analysis which we have documented, in that we can’t rely on and therefore did not report a direction of effect in our cumulative score calculation. When we do investigate certain relationship, we fall back to a posteriori knowledge from other empirical studies.

**4. Statistical Uncertainty: The MR estimates and binding affinity values both have standard errors, but their propagation through the final "side effect score" is unclear. How are uncertainties handled?**

We used parametric bootstrapping to capture uncertainty when calculating a score from multiple standard errors.

We have added this explanation to the methods section:

Parametric bootstrapping is a statistical approach to generating confidence intervals from resampled or simulated data.

Both $\bar{K_{i}M}$ (binding affinity estimate) and $\beta_{IV}$ (MR estimate) have standard errors, and to obtain the standard error for $S_{d,t}$ we used parametric bootstrap with 1000 iterations. This involves parametric re-sampling values for $K_{i}M$ and $\beta_{IV}$ to obtain bootstrap estimates of $S_{d,t}$. The final reported standard error of $S_{d,t}$ is the standard deviation of the 1000 boostrapped estimates. The code used to perform this calculation is in the GitHub repo here: <https://github.com/andrew-e/side-effect-score/blob/90b89d6b0a05b421104ba12f1d952cf125cd875a/R/calculate_side_effect_score.R#L40>.

**5. Figure Clarity: Some figures (e.g., Figure 3, cumulative drug scores) are difficult to interpret.**

Thank you for the suggestion. The figures have been altered in an attempt to provide better clarity. Namely, we have altered figure 3 to highlight the cumulative effect of each drug broken down by receptor, and figure 4 to highlight the comparative effect of each drug by side-effect.

## Reviewer 3:

We would like to thank reviewer 3 for their time and effort in reviewing our manuscript. We have responded to individual points inline.

**1. The statement “Genetic colocalization represents an additional criterion for causality…” may be misleading. Colocalization does not establish causality on its own but rather provides supportive evidence that two traits share a causal variant. The authors may consider rephrasing this to reflect that colocalization strengthens causal inference when combined with MR.**

Thank you for your response. We have updated the statement to better reflect your suggestion.

Genetic colocalization provides additional supportive genetic evidence by assessing whether two association signals are consistent with a shared causal variant.

**2. Although the authors state that the scores are best interpreted when comparing among drugs, some discrepancies (table 1) appear to contrast with known side-effect frequencies in the existing literatures. Further clarification would be helpful.**

Thanks for your comment. This is a pertinent point, as it highlights the current lack robust quantitative literature for the side-effects of drugs. Adverse event databases like SIDER (which we use), and the type of literature review that is provided by Stroup et. al(1) whom provide the less precise “+” categorical groupings are currently some of the best resources for known side effect profiles. Existing side-effect literature is unreliable due to known reasons, namely the lack of random control trails for medication, and inconsistent data collection and reporting. Compounding this issue, prediction of side effects is potentially biased by a number of mechanisms which we aim to be up front about in Figure 2.

Side-effect data collection occurs in two different axes of investigation, one is frequency and the other is severity. As an example, Stroup et al. combines the severity and frequency of side-effects and reports them together as a categorical variable (number of ‘+’s). Other times, which one is reported or focussed on is usually dependant on the side-effect. For example, Neutropenia is a serious but rare side-effect, so severity is usually discussed.

Attempts to improve this comparison have proven to be difficult. We tried to compare the calculated scores to the upper and lower bounded reported frequencies of side-effects collected by SIDER, however due to the amount of missing data, and limitations that frequency ranges alone are not a sufficient metric to compare our side-effect scores against, we have been unable to compare our results with any sort of gold standard.

We have attempted to be clear about the limitations of our quantitative prediction, namely outlining all known possible issues in Figure 2 of the manuscript. We have added additional text in the ‘Limitations’ section of ‘Discussion’ highlighting the lack of existing quantitative metrics in which we can meaningfully compare our results with.

**3a. The manuscript would benefit from a more detailed explanation of how SNP instruments were selected for the Wald ratio MR analysis. Specifically, did the authors use the per-SNP posterior probability of colocalization (SNP.PP.H4) to identify the top colocalizing variant for each pair? Clarifying this process would enhance both reproducibility and interpretability.**

Thank you for your response, the SNP instruments were not selected based on posterior probability information. They were selected in a 2-step process:

1. Genes were select from existing literature on the receptors that the drugs bind to (using both PDSP and Drugbank databases)
2. SNPs were chosen based off of the top hits as found in the eQTL datasets for each corresponding gene from step 1. This resulted in 1 SNP for eQTLGen, and between 1 and 4 SNPs from MetaBrain (depending on if each brain region had a different top hit).

We have provided a clearer explanation of the process of SNP IV selection in the ‘Methods’ section:

To identify genetic instruments, we first selected genes from existing literature on the receptors that the drugs bind to (using both PDSP and Drugbank databases). The top hits from eQTL databases (MetaBrain(17) and eQTLgen(20)) of each corresponding genes were selected as instrumental variables. This resulted in 1 SNP for eQTLGen, and between 1 and 4 SNPs from MetaBrain, depending on if each brain region had a different top hit.

**3b. Second, how did the authors ensure that the selected SNP instruments are not confounded by LD with variants that influence the outcome through pathways other than gene expression? A brief discussion of this would be valuable.**

Thank you for your comment. Colocalisation attempts to deal with the issue of LD. Horizontal vs. vertical pleiotropy is currently difficult to differentiate due low specificity of the instruments. This is a limitation which is common to all molecular MR studies.

To avoid confounding by LD, SNPs that are confounded by LD are dealt with in part by using colocalisation to pick apart the difference between MR results using SNP instruments that are merely showing correlated p-values, and those that share the same LD structure.

**4. The use of a relaxed colocalization threshold (H4 > 0.6, H3 < 0.1) deviates from the standard of H4 > 0.8. Can the authors justify this choice and provide any sensitivity analyses to assess how results change under different criteria?**

The colocalization threshold we use does deviate from the standard of H4 >= 0.8, however there is a relationship between colocalization scores that use data that has reached genome wide significance, and those that have not.

As this investigation focuses on a smaller subset of SNPs (number of receptors * number of IVs per receptor), multiple testing correction is lower than the standard genome wide significance threshold. Colocalization is sensitive to strength of the signal it is analysing. This has also been reflected in recent literature, as more published works are beginning to publish colocalization scores that are lower than the existing threshold of H4 >=0.8.

The issue with confounding by LD would lead to there being uncertainty about colocalization due to H3 and H4 both having high probabilities. However, where the threshold for H4 < 0.8, it is due to uncertainty between H4 and H2, in that colocalization is unsure if there is an association with the outcome. We have pre-screened for associations with the outcome which supports the case for H4.

We have provided additional information regarding the relationship between H4 posterior probability and minimum p-value of the extracted PheWAS region, which has been added as an additional supplementary material. As you can see, there is a significant relationship between those two values.

Please see ‘Text 1: Explanation of Colocalization Thresholds’ under ‘Supplementary Text and Figures’.

**5. Can the authors clarify the ancestry information of the eQTL, GWAS, and binding information datasets? If the datasets are drawn from different ancestries, it would be helpful to discuss the potential impact on instrument validity and generalizability of the results.**

The GWAS and eQTL datasets are either exclusively European ancestry, or a meta-analysis of individuals which of whom are majority European ancestry, and therefore would have a dominant role in the GWAS results. GWAS results that were primarily from a different ancestry were removed as a source.

Binding affinity data is an amalgamation of studies collected into the PDSP database, and therefore does not have a single source, however most binding affinity cell lines are derived from European populations. Binding affinities may change based on protein conformation, which is influenced by coding variants. If coding variants have different frequencies in different populations, binding affinities could change.

As the GWAS, eQTL, and binding affinity information is primarily derived from the same population, the results would be internally consistent. However, this does pose a risk that the results are not universally applicable. As the paper had no discussion on ancestry, we have updated the discussion section to include it, and identify a lack of diverse ancestry groups as a limitation. The methods section to specify the ancestry of the studies used.

The following has been added to the ‘Methods’ section:

GWAS and QTL datasets are either solely from European ancestry, or in the case of some GWASes are meta-analyses, of which 90% or more of the participants in the GWAS are from European ancestry. Binding affinity data is a cumulation of studies, however most binding affinity cell lines are derived from European populations. Although the analysis does not account for ancestral differences, the data used are ancestrally consistent.

The following has been added to the ‘Limitations’ section:

The analysis is also limited by ancestry. MetaBrain is a European only dataset, and eQTLGen is a majority European dataset. Although some of the GWAS summary statistics that were used from OpenGWAS were multi-ancestry, all had the majority of the samples used from European individuals and therefore the results of the GWAS were dominated by European individuals. Consequently, the analysis is internally consistent to provide relevant results, but we cannot generalize our findings to individuals from other ancestries.

**6. The analysis of scores derived from both on-target and off-target receptors appears somewhat limited in the manuscript.**

Thank you for your comment. We had attempted to highlight the difference between the two in the abstract and discussion, which investigates the primarily off-target mechanisms in which these side-effects occur. We have updated the figures to highlight the difference between on-target and off-target effects. We have also added additional information highlighting the difference between on-target and off-target receptors.

**Incorporating simulations to assess the behavior of the scoring calculation under controlled conditions—such as varying effect sizes, could provide stronger support for the reliability and interpretability of the score proposed in the manuscript.**

Thank you for your suggestion on providing some additional reliability by running simulations. Some of the aspects of the formula for calculating side-effect score are well covered in literature (Wald ratio, pKi as a measure of binding affinity, and using parametric bootstrapping). This is evidenced by the following 3D graph, where for each individual score, there is an expected relationship between Wald ratio effect size, pKi, and overall score. Each score is per receptor and drug, with no cumulation.

3D Visualisation of Drug Effect Scores

This leaves providing simulations on the cumulative effect of the score itself. We have altered the data in the repository that houses the score formula (<https://github.com/andrew-e/side-effect-score>) to understand how scores are altered if the effect of a one or more receptors are significantly altered, and if that in turn provides an oversized change in the overall score.

The two axes to investigate is binding affinity size and MR effect size.

Original Data

```
side_effect                         drug          score    receptors    pki_mean
White blood cell count decreased    aripiprazole  0.294            4       1.464
White blood cell count decreased    clozapine     0.823            5       2.961
White blood cell count decreased    olanzapine    0.237            5       3.081
White blood cell count decreased    paliperidone  0.104            4       1.166
White blood cell count decreased    quetiapine    0.442            3       0.830
White blood cell count decreased    risperidone   0.135            3       0.787
```

**Binding Affinity Changes**

All HTR6 values used the same original Wald ratio effect (0.02) with differing pKi binding affinities. When I remove HTR6 receptor from the results.

```
side_effect                         drug          score    receptors    pki_mean  change      %
White blood cell count decreased    aripiprazole  0.253            3       1.464   0.041   86.1
White blood cell count decreased    clozapine     0.647            4       2.961   0.176   78.6
White blood cell count decreased    olanzapine    0.164            4       3.081   0.073   69.2
White blood cell count decreased    paliperidone  0.082            3       1.166   0.022   78.8
White blood cell count decreased    quetiapine    0.390            2       0.830   0.052   88.2
White blood cell count decreased    risperidone   0.122            2       0.787   0.013   90.4
```

This shows a balanced and predictable relationship between the pKi value, number of receptors involved in the side effect. The larger the pKi value that was removed, the percentage change occurred, when factoring in the number of receptors the total score was involved in the calculation.

**Wald ratio changes**

If we keep all other values consistent and double the Wald ratio effect for the same receptor HTR6, with the dosage adjustment applied across each drug.

```
side_effect                      drug         score receptors. dosage_adj change      %
White blood cell count decreased aripiprazole 0.332         4        1.17  0.038    112
White blood cell count decreased clozapine    1.00          5        2.47  0.177    121
White blood cell count decreased olanzapine   0.311         5        1.00  0.074    131
White blood cell count decreased paliperidone 0.132         4        0.22  0.028    126
White blood cell count decreased quetiapine   0.492         3        4.68  0.050    111
White blood cell count decreased risperidone  0.147         3        0.31  0.012    108
```

This shows a balanced and predictable relationship between the pKi value, number of receptors involved in the side effect. The change in scores are more dependent on how much HTR6 individually impacted the score compared to other receptors, as well and how many receptors there are. We still see a proportional increase in total score

These simulations show linear relationships between altering both pKi and Wald ratio values and the final score, which is the desired effect of the formula.

**7. There are some typographical and grammatical errors throughout the manuscript (e.g., line 332). A careful proofreading pass is recommended prior to publication.**

Thank you for your feedback. We have proofread and updated the manuscript accordingly.

## References:

1.	Stroup TS, Gray N. Management of common adverse effects of antipsychotic medications. World Psychiatry. 2018 Oct;17(3):341–56.

2.	Aguet F, Brown AA, Castel SE, Davis JR, He Y, Jo B, et al. Genetic effects on gene expression across human tissues. Nature. 2017 Oct;550(7675):204–13.
