# Supplementary material for: Genetic inference of on-target and off-target side-effects of antipsychotic medications
Source: PLoS Genet. 2025 Jul 28;21(7):e1011793. doi: 10.1371/journal.pgen.1011793 (PMC12321077; doi:10.1371/journal.pgen.1011793)
Supplement: Attachment — Submitted filename: Response_to_Reviewers_auresp_2.docx [file pgen.1011793.s006.docx]

# Response to Reviewers

**Author response:** We thank the reviewers for the careful evaluation of the manuscript. We have taken all the comments into consideration for this resubmission. Below, we have a point-by-point response to each of the reviewer comments, and the corresponding changes are indicated in the manuscript as indented red text.

Reviewers 1 and 3 had no comments to address.

## Reviewer 2:

**The authors have addressed my comments from the first round comprehensively and made substantial improvements to the manuscript. I have a few minor points that may further strengthen the manuscript:**

**1. Tissue Relevance and Pleiotropy: While I appreciate the authors’ discussion around tissue-sharing of eQTLs and pleiotropy, I still encourage a clearer disclaimer in the abstract or introduction that the tissue-specific interpretations are limited. A short clarification that colocalization signals should not be interpreted as tissue-specific mechanisms would help prevent misinterpretation by readers unfamiliar with eQTL generalizability.**

Thank you for the suggestion. We have updated the text in the introduction about the limitations of tissue-specific and colocalization signals. Here is the following text that has been added:

While our study leverages tissue-specific eQTL data to explore potential mechanisms underlying antipsychotic side-effects, it is important to note that colocalization signals should not be interpreted as definitive evidence of tissue-specific mechanisms. Many eQTL signals are shared across tissues, and observed associations may reflect pleiotropic effects rather than tissue-specific pathways. We relied on the documented phenomena that cis eQTLs tend to be correlated and colocalizing across different tissues(13).

**2. Reproducibility: The authors now provide detailed methods and code access, which is excellent. I suggest also providing a minimal reproducible example (e.g., for one drug-side-effect pair) as a Jupyter or RMarkdown notebook in the GitHub repository to promote adoption by the community. I would also suggest releasing the data for the analysis if possible.**

Thank you for your suggestion, we have updated the GitHub repository with a more illustrative example, which includes 2 drugs and a series of side-effects over many receptors. We have included an RMarkdown notebook, and a corresponding html of the run notebook inside the repository. The reviewer can find the updates to the GitHub repository here: <https://github.com/andrew-e/side-effect-score>. Manuscript text has been updated to reflect the updates to the repository.

An example of the algorithm used to create these scores and a walkthrough of how it can be used in a R markdown file can be found at <https://github.com/andrew-e/side-effect-score>.

As for the real data for the analysis, this is already included in supplementary table 1.
