# Supplementary material for: Genetic inference of on-target and off-target side-effects of antipsychotic medications
Source: PLoS Genet. 2025 Jul 28;21(7):e1011793. doi: 10.1371/journal.pgen.1011793 (PMC12321077; doi:10.1371/journal.pgen.1011793)
Supplement: Peer review history

# Peer review: Decision Letter 0

**DOI:** 10.1371/journal.pgen.1011793.r001

**Authors:** Xiang Zhou, Xiaofeng Zhu

PGENETICS-D-25-00047

Genetic Inference of On-target and Off-target Side-effects of Antipsychotic Medications

PLOS Genetics

Dear Dr. Elmore,

Thank you for submitting your manuscript to PLOS Genetics. After careful consideration, we feel that it has merit but does not fully meet PLOS Genetics's publication criteria as it currently stands. Therefore, we invite you to submit a revised version of the manuscript that addresses the points raised during the review process.

Please submit your revised manuscript within 60 days Jun 03 2025 11:59PM. If you will need more time than this to complete your revisions, please reply to this message or contact the journal office at plosgenetics@plos.org. When you're ready to submit your revision, log on to https://www.editorialmanager.com/pgenetics/ and select the 'Submissions Needing Revision' folder to locate your manuscript file.

Please include the following items when submitting your revised manuscript:

* A rebuttal letter that responds to each point raised by the editor and reviewer(s). You should upload this letter as a separate file labeled 'Response to Reviewers'. This file does not need to include responses to any formatting updates and technical items listed in the 'Journal Requirements' section below.

* A marked-up copy of your manuscript that highlights changes made to the original version. You should upload this as a separate file labeled 'Revised Manuscript with Track Changes'.

* An unmarked version of your revised paper without tracked changes. You should upload this as a separate file labeled 'Manuscript'.

If you would like to make changes to your financial disclosure, competing interests statement, or data availability statement, please make these updates within the submission form at the time of resubmission. Guidelines for resubmitting your figure files are available below the reviewer comments at the end of this letter.

We look forward to receiving your revised manuscript.

Kind regards,

Xiang Zhou, Ph.D.

Academic Editor

PLOS Genetics

Xiaofeng Zhu

Section Editor

PLOS Genetics

Aimée Dudley

Editor-in-Chief

PLOS Genetics

Anne Goriely

Editor-in-Chief

PLOS Genetics

**Journal Requirements:**

1) Please ensure that the CRediT author contributions listed for every co-author are completed accurately and in full.

At this stage, the following Authors/Authors require contributions: Andrew R. Elmore, Aws Sadik, Lavinia Paternoster, Golam M. Khandaker, Tom R. Gaunt, and Gibran Hemani. Please ensure that the full contributions of each author are acknowledged in the "Add/Edit/Remove Authors" section of our submission form.

The list of CRediT author contributions may be found here: https://journals.plos.org/plosgenetics/s/authorship#loc-author-contributions

2) Please provide an Author Summary. This should appear in your manuscript between the Abstract (if applicable) and the Introduction, and should be 150-200 words long. The aim should be to make your findings accessible to a wide audience that includes both scientists and non-scientists. Sample summaries can be found on our website under Submission Guidelines:

https://journals.plos.org/plosgenetics/s/submission-guidelines#loc-parts-of-a-submission

3) Please upload all main figures as separate Figure files in .tif or .eps format. For more information about how to convert and format your figure files please see our guidelines: 

https://journals.plos.org/plosgenetics/s/figures

4) We have noticed that you have uploaded Supporting Information files, but you have not included a list of legends. Please add a full list of legends for your Supporting Information files after the references list.

5) Please ensure that the funders and grant numbers match between the Financial Disclosure field and the Funding Information tab in your submission form. Note that the funders must be provided in the same order in both places as well. State what role the funders took in the study. If the funders had no role in your study, please state: "The funders had no role in study design, data collection and analysis, decision to publish, or preparation of the manuscript.".

If you did not receive any funding for this study, please simply state: u201cThe authors received no specific funding for this work.u201d

**Reviewers' comments:**

Reviewer's Responses to Questions

**Comments to the Authors:**

**Please note here if the review is uploaded as an attachment.**

Reviewer #1: Genetic Inference of On-target and Off-target Side-effects of Antipsychotic Medications

PGENETICS-D-25-00047

Andrew R. Elmore et al

The is an interesting study with supportive evidence on the utilization of Mendelian Randomization for advancing our understanding of the side effects of commonly prescribed antipsychotics. Although, the utilization of MR for advancing pharmacogenomics research and implementation has been suggested in the literature. This manuscript provides convincing evidence at least for antipsychotics. Although in their conclusions the authors claim that this approach could be used to help inform drug choice for patients and clinicians, perhaps they need to specify the mechanisms of the clinical utility of their approach, especially in light of the stringent requiring of pharmacogenomics implementation.

I was tried to identify some sort of “a positive control” (a drug with a known side effect(s) that is mediated by a known genetic factor(s)” for the approach used by the authors. If they did, then the authors might want to clarify this.

The literature on the use of MR in drug repurposing and potential use in pharmacogenomics might benefit from more coverage and inclusion of the relevant published articles and reviews.

Comments on the potential utility of this approach for drugs used for the treatment of other common conditions such metabolic conditions and cancer might be useful for the readers.

Overall, I am supportive of accepting this manuscript in PLOS Genetics.

Reviewer #2: This manuscript introduces a methodology combining Mendelian randomization (MR) and drug binding affinity data to infer causal mechanisms underlying antipsychotic drug side effects. The authors report 36 side effects linked to 30 receptors (primarily off-target) across six antipsychotics. While the approach is innovative, methodological and interpretive limitations undermine the robustness and clinical applicability of the findings. Below are my specific comments:

Major comments:

1. The effect score of side effect per receptor is well-defined but lacks evaluation. Table 1 uses subjective "+" symbols to compare scores with existing literature, lacking quantitative alignment (e.g., statistical tests, correlation coefficients). This undermines the credibility of claimed corroborations. I believe a more systemically quantitative instead of qualitative evaluation will showcase the benefits of such definition of effect score. For example, scores can be compared to real-world side effect incidence (e.g., clinical trial data or adverse event databases).

2. The study would benefit from using pQTLs over eQTLs in MR due to the following reasons:

• pQTLs directly reflect genetic regulation of protein levels, which are more proximal to drug-receptor interactions and downstream phenotypic effects (e.g., side effects). Drugs act on proteins, not mRNA. Using pQTLs aligns with the pharmacological mechanism of antipsychotics (e.g., receptor binding affinity).

• eQTLs measure mRNA expression, which does not always correlate with protein abundance due to post-transcriptional regulation (e.g., translation efficiency, protein degradation). The authors themselves note that eQTL and pQTL directions disagree in ~30% of cases, undermining confidence in eQTL-based causal estimates.

Moreover, the study relies on brain-derived eQTLs (MetaBrain) for systemic side effects (e.g., neutropenia, blood pressure), which is biologically implausible. For example, neutropenia (low white blood cells) likely involves receptors in hematopoietic tissues (e.g., bone marrow), not brain. Blood pressure regulation involves vascular or renal tissues.

3. Interpretation of Binding Affinity and Drug Dosing. The study integrates binding affinity data (Ki values) into the score However, binding affinity alone does not dictate receptor activation. Some drugs act as partial agonists or inverse agonists, which could influence the interpretation of receptor-side-effect relationships.

4. Statistical Uncertainty: The MR estimates and binding affinity values both have standard errors, but their propagation through the final "side effect score" is unclear. How are uncertainties handled?

5. Figure Clarity: Some figures (e.g., Figure 3, cumulative drug scores) are difficult to interpret.

Reviewer #3: 1. The statement “Genetic colocalization represents an additional criterion for causality…” may be misleading. Colocalization does not establish causality on its own but rather provides supportive evidence that two traits share a causal variant. The authors may consider rephrasing this to reflect that colocalization strengthens causal inference when combined with MR.

2. Although the authors state that the scores are best interpreted when comparing among drugs, some discrepancies (table 1) appear to contrast with known side-effect frequencies in the existing literatures. Further clarification would be helpful.

3. The manuscript would benefit from a more detailed explanation of how SNP instruments were selected for the Wald ratio MR analysis. Specifically, did the authors use the per-SNP posterior probability of colocalization (SNP.PP.H4) to identify the top colocalizing variant for each pair? Clarifying this process would enhance both reproducibility and interpretability. Second, how did the authors ensure that the selected SNP instruments are not confounded by LD with variants that influence the outcome through pathways other than gene expression? A brief discussion of this would be valuable.

4. The use of a relaxed colocalization threshold (H4 > 0.6, H3 < 0.1) deviates from the standard of H4 > 0.8. Can the authors justify this choice and provide any sensitivity analyses to assess how results change under different criteria?

5. Can the authors clarify the ancestry information of the eQTL, GWAS, and binding information datasets? If the datasets are drawn from different ancestries, it would be helpful to discuss the potential impact on instrument validity and generalizability of the results.

6. The analysis of scores derived from both on-target and off-target receptors appears somewhat limited in the manuscript. Incorporating simulations to assess the behavior of the scoring calculation under controlled conditions—such as varying effect sizes, could provide stronger support for the reliability and interpretability of the score proposed in the manuscript.

7. There are some typographical and grammatical errors throughout the manuscript (e.g., line 332). A careful proofreading pass is recommended prior to publication.

**********

**Have all data underlying the figures and results presented in the manuscript been provided?**

Large-scale datasets should be made available via a public repository as described in the *PLOS Genetics* data availability policy , and numerical data that underlies graphs or summary statistics should be provided in spreadsheet form as supporting information.

Reviewer #1: Yes

Reviewer #2: None

Reviewer #3: Yes

**********

PLOS authors have the option to publish the peer review history of their article (what does this mean? ). If published, this will include your full peer review and any attached files.

If you choose “no”, your identity will remain anonymous but your review may still be made public.

**Do you want your identity to be public for this peer review?** For information about this choice, including consent withdrawal, please see our Privacy Policy .

Reviewer #1: No

Reviewer #2: No

Reviewer #3: No

[NOTE: If reviewer comments were submitted as an attachment file, they will be attached to this email and accessible via the submission site. Please log into your account, locate the manuscript record, and check for the action link "View Attachments". If this link does not appear, there are no attachment files.]

**Figure resubmission:**

While revising your submission, please upload your figure files to the Preflight Analysis and Conversion Engine (PACE) digital diagnostic tool, https://pacev2.apexcovantage.com/. PACE helps ensure that figures meet PLOS requirements. To use PACE, you must first register as a user. Registration is free. Then, login and navigate to the UPLOAD tab, where you will find detailed instructions on how to use the tool. If you encounter any issues or have any questions when using PACE, please email PLOS at figures@plos.org. Please note that Supporting Information files do not need this step. If there are other versions of figure files still present in your submission file inventory at resubmission, please replace them with the PACE-processed versions.

**Reproducibility:**

To enhance the reproducibility of your results, we recommend that authors of applicable studies deposit laboratory protocols in protocols.io, where a protocol can be assigned its own identifier (DOI) such that it can be cited independently in the future. Additionally, PLOS ONE offers an option to publish peer-reviewed clinical study protocols. Read more information on sharing protocols at https://plos.org/protocols?utm_medium=editorial-email&utm_source=authorletters&utm_campaign=protocols

# Author comment: Author response to Decision Letter 1

**DOI:** 10.1371/journal.pgen.1011793.r002

19 May 2025

Attachment Submitted filename: Response to Reviewers.docx

# Peer review: Decision Letter 1

**DOI:** 10.1371/journal.pgen.1011793.r003

**Authors:** Xiang Zhou, Xiaofeng Zhu

PGENETICS-D-25-00047R1

Genetic Inference of On-target and Off-target Side-effects of Antipsychotic Medications

PLOS Genetics

Dear Dr. Elmore,

Thank you for submitting your manuscript to PLOS Genetics. After careful consideration, we feel that it has merit but does not fully meet PLOS Genetics's publication criteria as it currently stands. Therefore, we invite you to submit a revised version of the manuscript that addresses the points raised during the review process.

Please submit your revised manuscript within 30 days Jul 22 2025 11:59PM. If you will need more time than this to complete your revisions, please reply to this message or contact the journal office at plosgenetics@plos.org. When you're ready to submit your revision, log on to https://www.editorialmanager.com/pgenetics/ and select the 'Submissions Needing Revision' folder to locate your manuscript file.

Please include the following items when submitting your revised manuscript:

* A rebuttal letter that responds to each point raised by the editor and reviewer(s). You should upload this letter as a separate file labeled 'Response to Reviewers'. This file does not need to include responses to formatting updates and technical items listed in the 'Journal Requirements' section below.

* A marked-up copy of your manuscript that highlights changes made to the original version. You should upload this as a separate file labeled 'Revised Manuscript with Track Changes'.

* An unmarked version of your revised paper without tracked changes. You should upload this as a separate file labeled 'Manuscript'.

If you would like to make changes to your financial disclosure, competing interests statement, or data availability statement, please make these updates within the submission form at the time of resubmission. Guidelines for resubmitting your figure files are available below the reviewer comments at the end of this letter.

We look forward to receiving your revised manuscript.

Kind regards,

Xiang Zhou, Ph.D.

Academic Editor

PLOS Genetics

Xiaofeng Zhu

Section Editor

PLOS Genetics

Aimée Dudley

Editor-in-Chief

PLOS Genetics

Anne Goriely

Editor-in-Chief

PLOS Genetics

**Journal Requirements:**

1) Your current Financial Disclosure states, "Yes ↳ Please add funding details. This work was supported by the National Institute for Health and Care Research (NIHR) Bristol Biomedical Research Centre; grant no: NIHR 203315. The recipients of this award are AE, GMK, LP, TRG, and GH. This work is also supported by grants from the Medical Research Council (MRC) to the MRC Integrative Epidemiology Unit at the University of Bristol (grant nos: MC_UU_00032/01, MC_UU_00032/03, and MC_UU_00032/06). The recipients of this award are AE, GMK, LP, TRG, and GH. ".

However, your funding information on the submission form indicates NIHR Bristol Biomedical Research Centre ; grant no: NIHR 203315. The recipient of this award is Dr. Tom R. Gaunt

This work is also supported by grants from the Medical Research Council (MRC) to the MRC Integrative Epidemiology Unit at the University of Bristol (grant nos: MC_UU_00032/01, MC_UU_00032/03, and MC_UU_00032/06). The recipient of this award is Dr. Tom R. Gaunt.

Please indicate by return email the full and correct funding information for your study and confirm the order in which funding contributions should appear. Please be sure to indicate whether the funders played any role in the study design, data collection and analysis, decision to publish, or preparation of the manuscript.

**Reviewers' comments:**

Reviewer's Responses to Questions

**Comments to the Authors:**

**Please note here if the review is uploaded as an attachment.**

Reviewer #1: The authors addressed my comment adequately.

Reviewer #2: The authors have addressed my comments from the first round comprehensively and made substantial improvements to the manuscript. I have a few minor points that may further strengthen the manuscript:

1. Tissue Relevance and Pleiotropy: While I appreciate the authors’ discussion around tissue-sharing of eQTLs and pleiotropy, I still encourage a clearer disclaimer in the abstract or introduction that the tissue-specific interpretations are limited. A short clarification that colocalization signals should not be interpreted as tissue-specific mechanisms would help prevent misinterpretation by readers unfamiliar with eQTL generalizability.

2. Reproducibility: The authors now provide detailed methods and code access, which is excellent. I suggest also providing a minimal reproducible example (e.g., for one drug-side-effect pair) as a Jupyter or RMarkdown notebook in the GitHub repository to promote adoption by the community. I would also suggest releasing the data for the analysis if possible.

Reviewer #3: The authors have addressed my comments.

**********

**Have all data underlying the figures and results presented in the manuscript been provided?**

Large-scale datasets should be made available via a public repository as described in the *PLOS Genetics* data availability policy , and numerical data that underlies graphs or summary statistics should be provided in spreadsheet form as supporting information.

Reviewer #1: Yes

Reviewer #2: **No: **

Reviewer #3: Yes

**********

PLOS authors have the option to publish the peer review history of their article (what does this mean? ). If published, this will include your full peer review and any attached files.

If you choose “no”, your identity will remain anonymous but your review may still be made public.

**Do you want your identity to be public for this peer review?** For information about this choice, including consent withdrawal, please see our Privacy Policy .

Reviewer #1: No

Reviewer #2: No

Reviewer #3: No

**Figure resubmission:**

While revising your submission, please upload your figure files to the Preflight Analysis and Conversion Engine (PACE) digital diagnostic tool, https://pacev2.apexcovantage.com/. PACE helps ensure that figures meet PLOS requirements. To use PACE, you must first register as a user. Registration is free. Then, login and navigate to the UPLOAD tab, where you will find detailed instructions on how to use the tool. If you encounter any issues or have any questions when using PACE, please email PLOS at figures@plos.org. Please note that Supporting Information files do not need this step. If there are other versions of figure files still present in your submission file inventory at resubmission, please replace them with the PACE-processed versions.

**Reproducibility:**

To enhance the reproducibility of your results, we recommend that authors deposit laboratory protocols in protocols.io, where a protocol can be assigned its own identifier (DOI) such that it can be cited independently in the future. Additionally, PLOS ONE offers an option to publish peer-reviewed clinical study protocols. Read more information on sharing protocols at https://plos.org/protocols?utm_medium=editorial-email&utm_source=authorletters&utm_campaign=protocols

# Author comment: Author response to Decision Letter 2

**DOI:** 10.1371/journal.pgen.1011793.r004

27 Jun 2025

Attachment Submitted filename: Response_to_Reviewers_auresp_2.docx

# Editor report: Decision Letter 2

**DOI:** 10.1371/journal.pgen.1011793.r005

**Authors:** Xiang Zhou, Xiaofeng Zhu

Dear Dr Elmore,

We are pleased to inform you that your manuscript entitled "Genetic Inference of On-target and Off-target Side-effects of Antipsychotic Medications" has been editorially accepted for publication in PLOS Genetics. Congratulations!

Before your submission can be formally accepted and sent to production you will need to complete our formatting changes, which you will receive in a follow up email. Please be aware that it may take several days for you to receive this email; during this time no action is required by you. Please note: the accept date on your published article will reflect the date of this provisional acceptance, but your manuscript will not be scheduled for publication until the required changes have been made.

Once your paper is formally accepted, an uncorrected proof of your manuscript will be published online ahead of the final version, unless you’ve already opted out via the online submission form. If, for any reason, you do not want an earlier version of your manuscript published online or are unsure if you have already indicated as such, please let the journal staff know immediately at plosgenetics@plos.org.

In the meantime, please log into Editorial Manager at https://www.editorialmanager.com/pgenetics/, click the "Update My Information" link at the top of the page, and update your user information to ensure an efficient production and billing process. Note that PLOS requires an ORCID iD for all corresponding authors. Therefore, please ensure that you have an ORCID iD and that it is validated in Editorial Manager. To do this, go to ‘Update my Information’ (in the upper left-hand corner of the main menu), and click on the Fetch/Validate link next to the ORCID field.  This will take you to the ORCID site and allow you to create a new iD or authenticate a pre-existing iD in Editorial Manager.

If you have a press-related query, or would like to know about making your underlying data available (as you will be aware, this is required for publication), please see the end of this email. If your institution or institutions have a press office, please notify them about your upcoming article at this point, to enable them to help maximise its impact. Inform journal staff as soon as possible if you are preparing a press release for your article and need a publication date.

Thank you again for supporting open-access publishing; we are looking forward to publishing your work in PLOS Genetics!

Yours sincerely,

Xiang Zhou, Ph.D.

Academic Editor

PLOS Genetics

Xiaofeng Zhu

Section Editor

PLOS Genetics

Aimée Dudley

Editor-in-Chief

PLOS Genetics

Anne Goriely

Editor-in-Chief

PLOS Genetics

www.plosgenetics.org

Twitter: @PLOSGenetics

----------------------------------------------------

Comments from the reviewers (if applicable):

----------------------------------------------------

**Data Deposition**

If you have submitted a Research Article or Front Matter that has associated data that are not suitable for deposition in a subject-specific public repository (such as GenBank or ArrayExpress), one way to make that data available is to deposit it in the Dryad Digital Repository . As you may recall, we ask all authors to agree to make data available; this is one way to achieve that. A full list of recommended repositories can be found on our website .

The following link will take you to the Dryad record for your article, so you won't have to re‐enter its bibliographic information, and can upload your files directly: 

http://datadryad.org/submit?journalID=pgenetics&manu=PGENETICS-D-25-00047R2

More information about depositing data in Dryad is available at http://www.datadryad.org/depositing. If you experience any difficulties in submitting your data, please contact help@datadryad.org for support.

Additionally, please be aware that our data availability policy  requires that all numerical data underlying display items are included with the submission, and you will need to provide this before we can formally accept your manuscript, if not already present.

----------------------------------------------------

**Press Queries**

If you or your institution will be preparing press materials for this manuscript, or if you need to know your paper's publication date for media purposes, please inform the journal staff as soon as possible so that your submission can be scheduled accordingly. Your manuscript will remain under a strict press embargo until the publication date and time. This means an early version of your manuscript will not be published ahead of your final version. PLOS Genetics may also choose to issue a press release for your article. If there's anything the journal should know or you'd like more information, please get in touch via plosgenetics@plos.org .

# Editor report: Acceptance letter

**DOI:** 10.1371/journal.pgen.1011793.r006

**Authors:** Xiang Zhou, Xiaofeng Zhu

PGENETICS-D-25-00047R2

Genetic Inference of On-target and Off-target Side-effects of Antipsychotic Medications

Dear Dr Elmore,

We are pleased to inform you that your manuscript entitled "Genetic Inference of On-target and Off-target Side-effects of Antipsychotic Medications" has been formally accepted for publication in PLOS Genetics! Your manuscript is now with our production department and you will be notified of the publication date in due course.

The corresponding author will soon be receiving a typeset proof for review, to ensure errors have not been introduced during production. Please review the PDF proof of your manuscript carefully, as this is the last chance to correct any errors. Please note that major changes, or those which affect the scientific understanding of the work, will likely cause delays to the publication date of your manuscript.

Soon after your final files are uploaded, unless you have opted out or your manuscript is a front-matter piece, the early version of your manuscript will be published online. The date of the early version will be your article's publication date. The final article will be published to the same URL, and all versions of the paper will be accessible to readers.

Thank you again for supporting PLOS Genetics and open-access publishing. We are looking forward to publishing your work!

With kind regards,

Anita Estes

PLOS Genetics

On behalf of:

The PLOS Genetics Team

Carlyle House, Carlyle Road, Cambridge CB4 3DN | United Kingdom

plosgenetics@plos.org | +44 (0) 1223-442823

plosgenetics.org | Twitter: @PLOSGenetics
